# Search for Chirality in Hydrogenated Magnesium Nanosilicates: A DFT and TD-DFT Investigation

**DOI:** 10.1021/acs.jpca.3c06521

**Published:** 2024-04-30

**Authors:** Kamil B. Stelmach, Catherine A. Dukes, Robin T. Garrod

**Affiliations:** † Department of Chemistry, University of Virginia, Charlottesville, Virginia 20904, United States; ‡ Laboratory for Astrophysics and Surface Physics, Department of Materials Science and Engineering, University of Virginia, Charlottesville, Virginia 22904, United States; § Department of Astronomy, University of Virginia, Charlottesville, Virginia 22904, United States

## Abstract

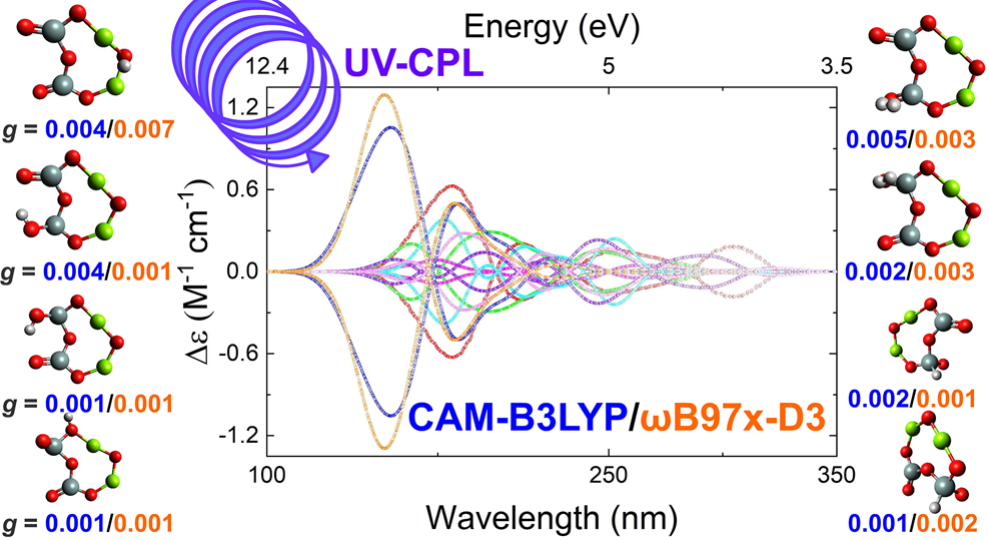

The formation of silicate grains in the interstellar medium (ISM), especially those containing chiral surfaces such as clinopyroxenes, is poorly understood. Moreover, silicate interactions with various forms of hydrogen–proton (H^+^), neutral H (HI), and molecular hydrogen (H_2_) are of high importance as hydrogen comprises >90% of the ISM gas budget, and these species play important roles in the formation of new molecules in space. Furthermore, silicate surfaces catalyze the formation of H_2_ in the interstellar medium formed on dust grain surfaces by H–H association. The technical difficulty of *in situ* laboratory investigations of nanosilicate nucleation using astrophysically relevant environmental conditions makes computational chemistry a useful tool for studying potential nanosilicate structures. Furthermore, chiral surfaces interacting with chiral organic molecules could serve as templates that lead to the enantiomeric excess of l-amino acids and d-polyols detected in carbonaceous meteorites. However, in order for this effect to take place, an excess of one chiral form of a mineral is required to break the symmetry. This symmetry-breaking event could have been due to the asymmetric absorption of circularly polarized light by the nanosilicate as it traverses star-forming regions. We investigate this possibility using a metastable chiral form of an enstatite dimer as an input for density functional theory (DFT) and time-dependent (TD)-DFT calculations to obtain various properties and circular dichroism spectra. All in all, twenty-six magnesium nanosilicate structures were studied using varying degrees of hydrogenation: none, with HI, with H^+^, and with H_2_. The HSE06/aug-cc-pVQZ level of theory was used for the DFT calculations. TD-DFT calculations utilized the CAM-B3LYP/cc-pVQZ and ωB97X-D3/cc-pVQZ functional and basic set pairings. Results show that (1) all twenty-six structures have absorption bands that fall within the 0.6–28.3 μm range available with the newly launched James Webb Space Telescope and (2) there is a small enantioselective effect by UV-CPL on the eight chiral enstatite dimers (predicted *g*-values of up to 0.007). While the observed effect is small, it opens up the possibility that it is the inorganic material that becomes enantiomerically biased by UV-CPL, driving chiral enhancements in meteoric organic molecules.

## 1 Introduction

Over 300 molecules have been found in interstellar space using microwave and infrared observational methods,^1^ where most of these species are relatively small organic molecules. Of these species, only one organic molecule, propylene oxide (CH_3_CHCH_2_O), has been identified as chiral.^2^ However, additional chiral organic molecules are posited to exist due to their presence in carbonaceous meteorites, which consist of various classes of amino acids, including α-amino acids, the building blocks of proteins, and sugar alcohols, related to the many sugars used in various metabolic processes or biological structures.^3^ Both of these classes of biomolecules, the α-amino acids and sugar alcohols, show an enantiomeric excess in the same directions as used by life (left-handed for amino acids and right-handed for sugars), despite abiotic reactions usually producing racemic mixtures. The origin of this symmetry-breaking mechanism remains unknown.

Silicon-containing molecules and minerals have also been identified in interstellar medium (ISM). These gas-phase Si molecules include SiO,^4^ SiS,^5^ SiC,^6^ SiN,^7^ SiCN,^8^ SiNC,^8^ SiCSi,^9^ SiC_3_,^10^ SiH_4_,^11^ SiC_4_,^12^ SiH_3_CN,^13,14^ and CH_3_SiH_3_.^14^ Solid-phase, bulk silicates such as enstatite, forsterite, and quartz—in both amorphous and crystalline form—have also been identified in regions from molecular clouds to the diffuse ISM to protoplanetary discs and stellar nebulae.^15,16^ Bulk enstatite has already been found around planetary nebulae (e.g., NGC 6302, colloquially the Butterfly Nebula),^17^ circumstellar envelopes around young Herbig Ae/Be stars,^15,16,18^ and main sequence stars like HD165014.^19^

The formation process that produces crystalline minerals is unknown, and the exact mechanisms have been a recent topic of debate (20 and references therein,^21,22^). Silicate synthesis can be roughly divided into either bottom-up mechanisms, where grains are built up from small nanosilicate molecules containing only a few atoms, or top-down mechanisms, where larger agglomerations of amorphous material are annealed and crystallized to form grains in a protoplanetary disk or solar nebula.^15,16^ The former is imagined as a two-step process with (1) the nucleation of growth centers and (2) subsequent local rearrangement to crystallize amorphous material or the addition of atoms to the crystal lattice.^23^

A few laboratory^22−24^ and computational studies^20,21,25−29^ have investigated the formation of bulk minerals from small nanosilicates or the properties of such nanosilicates, although not all were conducted under conditions relevant to a protoplanetary disk (PPD) or the interstellar medium (ISM). Laboratory experiments are limited, largely due to the difficulty of studying gas-phase grain formation in an astrophysically relevant laboratory setting, which must compensate for extremely low molecular densities, short lifetimes, and cryogenic gas-phase temperatures, thus requiring complicated optical detection methodologies (e.g., cavity ringdown spectroscopy). Nagahara et al.^23^ showed that bulk quartz minerals condense before enstatite-like silicate dust, with the latter remaining mostly amorphous in ISM-like conditions but crystallizing quickly in PPD-like conditions. Guiu et al.^30^ especially have shown that the formation of monomers with pyroxene stoichiometry is possible where nanosilicates quickly cool and persist, not forming a bulk mineral, and that molecular oxygen is attracted to the nanosilicate structures. Computational chemistry has proven to be synergistic with laboratory measurements and remains a powerful tool in exploring gas-phase formation of silicate grains, including the magnesium-rich inosilicate enstatite (MgSiO_3_),^20,21,29−31^ an astrophysically relevant mineral that lends its name to E-type meteorites and asteroids.

Bromley et al.^20^ studied the reaction chemistry of various nanosilicate grains and derived a reaction network containing seven steps to build up an enstatite dimer using density functional theory (DFT) starting with simple reagents like H_2_O, Mg, and SiO. Valencia et al.^21^ expanded on this, studying the physical properties of Mg-containing silicates using CCSD(T)-F12 calculations to include enstatite and forsterite (Mg_2_SiO_4_) monomers and dimers with the goal of obtaining infrared spectroscopic information, noting that these nanosilicate structures are highly detectable due to their large dipole moments. Escatllar et al.^29^ used computational tools to predict the IR spectra and energy properties of nanosilicates containing both olivine and pyroxene stoichiometries, showing that the spectra of nanosilicates are blue-shifted in comparison to their larger forms, while Guiu and Bromley^30^ suggest that the heat capacities of nanosilicates have previously been overestimated, increasing their detectability and making them potential targets for the new James Webb Space Telescope (JWST). Indeed, Zeegers et al.^31^ predict that JWST is capable of detecting percentages as small as a 3% nanosilicate fraction around O8 V or carbon-rich Wolf–Rayet stars using JWST’s mid-infrared instrument (MIRI).

Hydrogen is the most common element in the universe, comprising ∼90% of the gas in the ISM, where it is available to interact with Mg-containing nanosilicates in various forms (e.g., HI, H^+^, H_2_) in addition to H_3_^+^, which is responsible for many astrochemical reactions.^32^ Neutral hydrogen, HI, appears at various temperatures and pressures, as demonstrated by the observation of its 21 cm emission line, which has been identified in regions throughout the ISM.^33^ Protons are abundant as cosmic rays,^34^ as well as solar and stellar winds,^35^ and within regions of shock-heated plasmas.^36^ H_2_ is ubiquitous across the universe, appearing as a common component of molecular clouds and solar nebulae.^37,38^ Importantly, the formation of H_2_ is thought to occur on silicate grains by recombination of gas-phase adsorbed H,^25,28,39−42^ a process recently investigated on enstatite nanosilicates.^26,28^ The hydrogenation of nanosilicates is important to understand relevant gas and surface interactions.

We note that the mineral enstatite has two common crystallographic phases. On Earth, enstatite is predominantly found as an orthopyroxene (opx) without chiral faces. However, on other planetary bodies^43,44^ and around circumstellar, protoplanetary, and debris disks,^15,16^ enstatite is found as a clinopyroxene (cpx), which has several chiral faces, including the cleavage planes: ⟨100⟩ and ⟨110⟩.^45^ The other aforementioned minerals, namely, quartz and olivine, also have chiral faces,^46^ though quartz is mostly amorphous in the ISM and olivine’s chiral faces are phase specific.

Chiral mineral surfaces have been previously found to enhance the presence of certain amino acid enantiomers in an aqueous setting,^45−47^ with computational studies on quartz or calcite carried out in support of this hypothesis.^48−51^ In a vacuum environment such as the ISM, laboratory experiments have demonstrated that high-index metals with chiral surfaces can be enantioselective for a variety of chiral organic species, including propylene oxide, various amino acids, tartaric acid, and glycidol.^52−56^ However, no initial symmetry-breaking event for chiral minerals has been identified on Earth, where there is an equal abundance of both enantiomorphs of chiral surfaces.^57^

The presence of clinoenstatite in the Solar System and ISM, along with the detection of enantiomeric asymmetries in carbonaceous meteorite organics, allows for the re-examination of chiral surfaces as an explanation for biomolecular homochirality. Asymmetry in chiral mineral surfaces can disrupt the symmetry of chiral organic molecules, where a surface may act as a template for the preferential enhancement of one enantiomer over another during adsorption/desorption events. However, a mechanism for symmetry breakage within the minerals themselves is required; Yeom et al.^58^ showed that the twist direction of synthesized right- and left-handed twisted nanoribbons of CdTe nanoparticles can be influenced by circularly polarized light (CPL). CPL has also been found in interstellar space in the Orion Nebula.^59^ Thus, identifying chiral nanosilicates, potential building blocks for chiral mineral structures, is of high importance to test this hypothesis.

While Bromley et al.^20^ and Valencia et al.^21^ showed putative structures for enstatite and forsterite monomers and dimers, none of these structures were inherently chiral, nor did they examine the effects of hydrogenation on structures of this size. Here, we use a combination of density functional theory (DFT^60^) and time-dependent DFT (TD-DFT^61^) techniques to study hydrogenated versions of two Valencia et al.^21^ structures and a two-dimensional chiral conformer of the dimer. Since one of the hydrogenated versions of this chiral conformer was three-dimensionally chiral, ECD calculations were performed to explore whether CPL can preferentially interact with one of the enantiomers.

## 2 Computational Details

### 2.1 DFT Calculations

**Figure 1:**
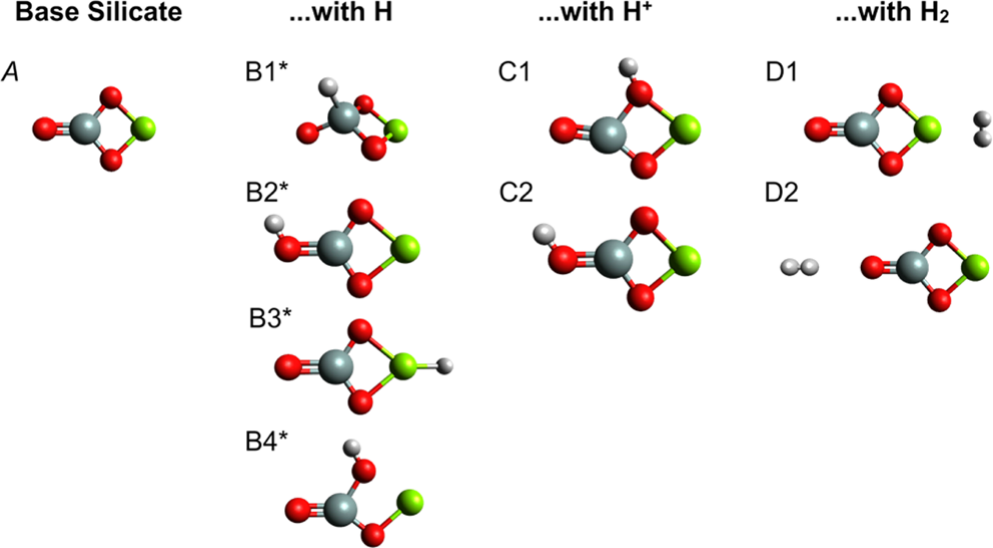
Optimized monomeric nanosilicate structures with and without hydrogenation (HI, H^+^, and H_2_). Oxygen atoms are red, silicon atoms are gray, magnesium atoms are green, and hydrogen atoms are white. The Valencia et al. (2020) molecule structure A is italicized. Structures hydrogenated with neutral hydrogen (B1–4) are open-shell structures and are indicated with a star (*). None of the monomer structures were chiral.

**Figure 2:**
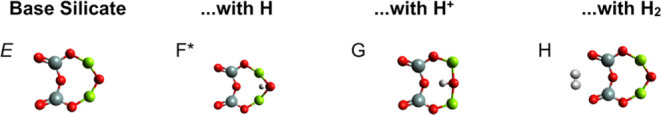
Optimized achiral dimeric nanosilicate structures with and without hydrogenation (HI, H^+^, and H_2_). Oxygen atoms are red, silicon atoms are gray, magnesium atoms are green, and hydrogen atoms are white. The Valencia et al. (2020) molecule structure B is italicized. Structure F is an open-shell structure and is indicated with a star (*).

**Figure 3:**
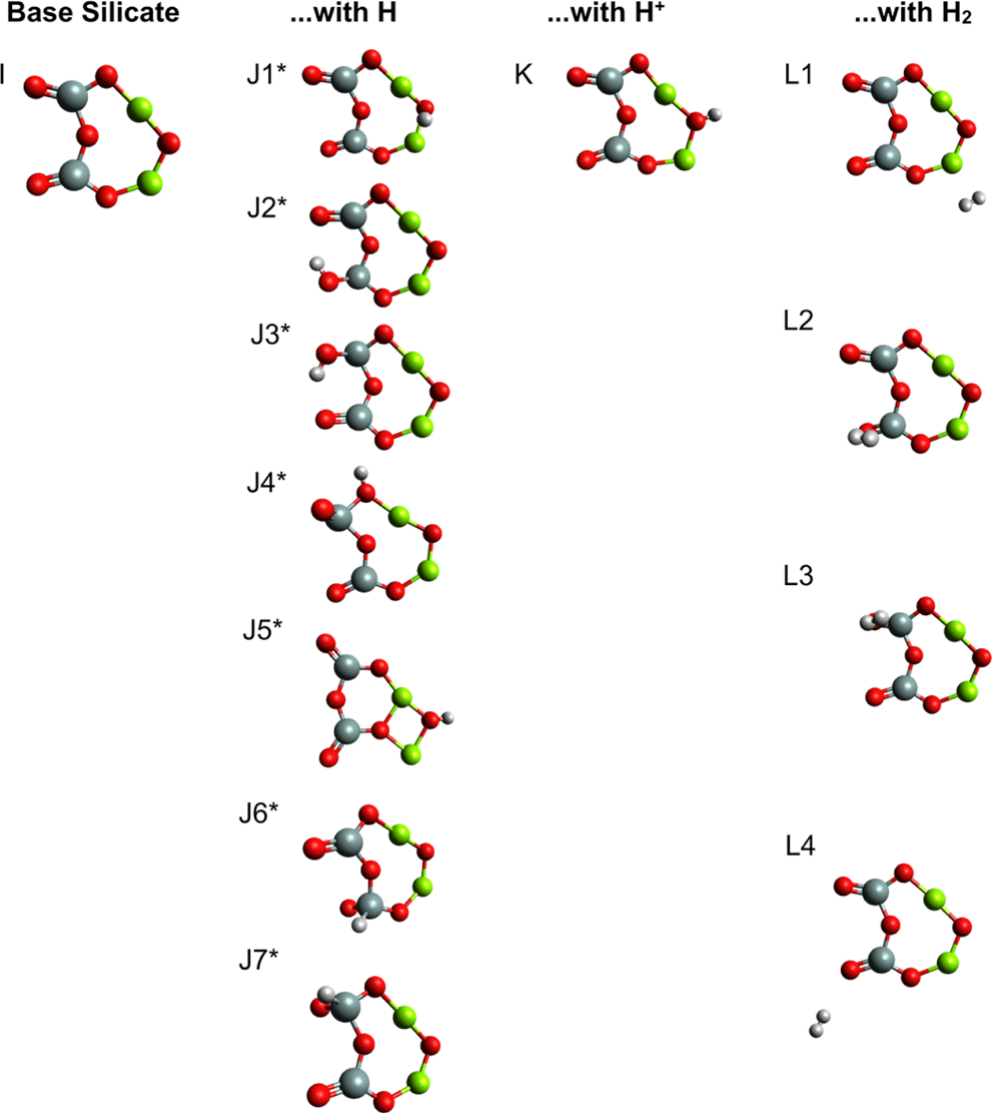
Optimized chiral dimeric nanosilicate structures with and without hydrogenation (HI, H^+^, and H_2_). Oxygen atoms are red, silicon atoms are gray, magnesium atoms are green, and hydrogen atoms are white. The J structures are open-shell structures and are indicated with a star (*).

Density functional theory (DFT) is a computational quantum chemistry technique that is used to obtain the accurate physical properties of molecules at a fraction of the computational cost of higher levels of theory like MP2 or CCSD calculations.^62^ In this work, Avogadro 1.2.0—a molecular editing and viewing program—was used to create initial monomer and dimer nanosilicate structures prior to optimization.^63^ Gaussian16^64,65^ was used for all of the DFT calculations of geometries, frequencies, and energies of the studied molecules. Figures 1–3 show the geometrically optimized versions of the studied enstatite nanosilicate structures, where one monomer (structure A, Figure 1) and one achiral dimer (structure E, Figure 2) were previously studied by Valencia et al.^21^ We chose this specific dimer nanosilicate for its initially low symmetry, which enhances the potential for structural variation through hydrogenation. The bare enstatite monomer and dimer structures are shown (Figures 1–3), along with hydrogenated versions of the base structures with neutral hydrogen (H), a proton (H^+^), and molecular hydrogen (H_2_). Adsorbates were placed in systematic locations in the plane and a plane above the molecules, as shown in Figure S1. The presented structures are the final optimized structures that converged with the DFT calculations.

To select the optimal DFT functionals for our calculations, we benchmarked three hybrid DFT functionals, namely, B3LYP, B3PW91, and HSE06,^66−70^ against the CCSD(T)-F12/cc-pVTZ-F12 calculations performed by Valencia et al.^21^ on the enstatite monomer (geometry and frequency calculations). The functionals were chosen based on computational studies that indicate these functionals can predict silicate properties well.^71−73^ Several basis sets were used for the comparison, including cc-pVQZ,^74−76^ aug-cc-pVQZ,^74−77^ and DGTZVP.^78,79^

After benchmarking the bare monomer structure and spectra (Valencia et al.^21^), all structures were subsequently optimized using the HSE06/aug-cc-pVQZ level of theory using very tight convergence parameters (root-mean-square force criterion set to 10^–6^) and super fine grid (175,974 points for first row atoms and 250,974 points for atoms in the second row and beyond). Energy calculations were conducted using the same level of theory. For the open-shell H-hydrogenated chiral enstatite dimer (J structures in Figure 3), the expectation value of the total spin ⟨*S*^2^⟩ is approximately equal to *s*(*s*+1) where *s* is half of the number of unpaired electrons (0.75 expected). The calculated expectation values for structures J1, J2, J3, J4, J5, J6, and J7 are 0.758374, 0.7520, 0.7521, 0.7538, 0.7503, 0.7538, and 0.7537, respectively.

### 2.2 TD-DFT Calculations

Time-dependent (TD)-DFT calculations provide solutions to the time-dependent Schrödinger equation for a many-bodied molecular system in the presence of time-varying potentials and fields. Orca Version 4.2.1^80,81^ was used for the TD-DFT calculations. While Gaussian16 does perform TD-DFT calculations, the algorithms used are not suitable for open-shell structures such as the neutral H-enstatite nanosilicates (second column of structures in Figures 1–3). We used two long-range functionals commonly used in TD-DFT calculations,^82^ CAM-B3LYP^83,84^ and ωB97X-D3,^85^ for our calculations with the cc-pVQZ basis set, selected due to its large size and minimal differences between it and the augmented version in test calculations. UV–vis spectra were calculated for all of the bare and hydrogenated enstatite monomer and dimer structures (Figures 1–3). An electronic circular dichroism spectrum was calculated for all of the three-dimensional chiral enstatite dimers (structures J1-J4, J6, J7, L2, and L3) to identify potential chiral asymmetries. A total of 60 excited states were calculated for all of the structures, which included both singlet and triplet states where appropriate. The RIJCOSX algorithm^86^ was used for all TD-DFT calculations.

## 3 Results and Discussion

### 3.1 HSE06/aug-cc-pVQZ Optimization and Frequency Calculations

#### 3.1.1 Geometrical Optimization

The bond lengths and angles for the geometrical optimizations for each monomer and dimer (Figures 1–3) are summarized in Tables 1, 2, and 3. Tables S6–S31 contain the MOL2 text file information about each structure. The bare monomer and achiral dimer enstatite structures (Figure 1A,E), both exhibiting *C*_2*V*_ symmetry, are consistent with the Valencia et al.^21^ results. The addition of neutral hydrogen, proton, or molecular hydrogen has a great effect on each structure type’s bond lengths and angles. All calculations were performed with the hydrogen species being placed in systematic locations at the plane of each molecule and above (see Figure S1), then allowing the H^+^/H/H_2_ to migrate. Not all of the tested positions resulted in a unique converged structure.

**Table 1:** Monomer Bond Lengths and Angles Calculated with HSE06/aug-cc-pVQZ for Figure 1

structure A monomer (MgSiO_3_)	bond lengths (Å)	Mg–Si	2.416
		O=Si	1.518
		Si–O	1.637
	bond angles (deg)	O=Si–O	129.9
		Si–O–Mg	87.29
structure B2 monomer with neutral hydrogen (MgSiO_3_–H)	bond lengths (Å)	Mg–Si	2.498
		O–H	0.959
		O=Si	1.608
		Si–O *cis* H	1.561
		Si–O *trans* H	1.557
	bond angles (deg)	O=Si–O *cis* H	125.5
		O=Si–O *trans* H	123.4
		H–O=Si	117.1
		Si–O–Mg *cis* H	85.76
		Si–O–Mg *trans* H	86.12
structure C2 monomer with proton (MgSiO_3_–H^+^)	bond lengths (Å)	Mg–Si	2.408
		O–H	0.961
		O=Si	1.575
		Si–O *cis* H	1.581
		Si–O *trans* H	1.577
	bond angles (deg)	O=Si–O *cis* H	129.6
		O=Si–O *trans* H	124.7
		H–O=Si	124.2
		Si–O–Mg *cis* H	86.1
		Si–O–Mg *trans* H	86.4
structure D1 monomer with molecular hydrogen (MgSiO_3_–H_2_)	bond lengths (Å)	H–H	0.756
		Mg–H	2.188
		Mg–Si	2.420
		O=Si	1.519
		Si–O	1.635
	bond angles (deg)	O=Si–O	129.8
		Si–O–Mg	87.37
		O–Mg–H	127.6

**Table 2:** Achiral Dimer Bond Lengths and Angles Calculated with HSE06/aug-cc-pVQZ for Figure 2

structure E achiral dimer (Mg_2_Si_2_O_6_)	bond lengths (Å)	Mg–Si	3.000
		O=Si	1.511
		Si-OMg	1.586
		SiO-Mg	1.841
		Mg-OMg	1.795
	bond angles (deg)	O=Si-OMg	131.3
		O=Si-OSi	124.4
		Si–O–Mg	122.0
		Mg–O–Mg	116.8
		Si–O–Si	148.2
structure F achiral dimer with nutral hydrogen (Mg_2_Si_2_O_6_–H)	bond lengths (Å)	Mg–Si	2.932
		Mg–H	1.937
		O=Si	1.510
		Si-OMg	1.584
		SiO-Mg	1.844
		Mg-OMg	1.880
	bond angles (deg)	O=Si-OMg	132.5
		O=Si-OSi	124.3
		Si–O–Mg	117.4
		Mg–O–Mg	100.4
		Si–O–Si	143.8
		Si_2_O-Mg_2_O-H	65.15
		SiO–Mg-H	127.7
structure G achiral dimer with proton (Mg_2_Si_2_O_6_–H^+^)	bond lengths (Å)	Mg–Si	3.111
		H-OMg_2_	1.003
		O=Si	1.504
		Si-OMg	1.593
		SiO-Mg	1.803
		Mg-OMg	1.898
	bond angles (deg)	O=Si-OMg	132.4
		O=Si-OSi	124.8
		Si–O–Mg	132.6
		Mg–O–Mg	172.9
		Si–O–Si	143.6
		Si_2_O-Mg_2_O-H	0.0
		Si-OSi_2_–H	108.2
structure H achiral dimer with molecular hydrogen (Mg_2_Si_2_O_6_–H_2_)	bond lengths (Å)	Mg–Si	3.004
		H-OSi_2_	3.480
		H–H	0.746
		O=Si	1.511
		Si-OMg	1.586
		SiO-Mg	1.841
		Mg-OMg	1.795
	bond angles (deg)	O=Si-OMg	131.0
		O=Si-OSi	124.5
		Si–O–Mg	122.3
		Mg–O–Mg	116.9
		Si–O–Si	148.8
		H-OSi_2_–Si	72.46
		Si_2_O-Mg_2_O-H	134.4

**Table 3:** Chiral Dimer Bond Lengths and Angles Calculated with HSE06/aug-cc-pVQZ for Figure 3

structure I chiral dimer (Mg_2_Si_2_O_6_)	bond lengths (Å)	Mg–Si	2.754	3.079
		O=Si	1.509	1.510
		Si-OMg	1.581	1.581
		SiO-Mg	1.877	1.840
		Mg-OMg	1.807	1.788
	bond angles (deg)	O=Si-OMg	136.1	131.5
		O=Si-OSi	124.6	122.7
		Si–O–Mg	105.3	128.2
		Mg–O–Mg	113.1	
		Si–O–Si	140.7	
structure J5 chiral dimer with neutral hydrogen (Mg_2_Si_2_O_6_–H)	bond lengths (Å)	Si-OSi	1.614	1.652
		O=Si	1.511	1.512
		Si-OMg	1.612	1.581
		SiO-Mg	1.997	1.843
		Mg-OMg	1.996	1.886
		O–H	0.953	
	bond angles (deg)	O=Si-OMg	125.3	131.2
		O=Si-OSi	126.0	120.2
		Si–O–Mg	140.5	127.7
		Mg–O–Mg	94.9	
		Si–O–Si	142.2	
		Si_2_O-Mg_2_O-H	161.7	
		SiO–Mg-H	98.6	100.4
structure K chiral dimer with proton (Mg_2_Si_2_O_6_–H^+^)	bond lengths (Å)	Mg–Si	2.639	3.221
		O=Si	1.501	1.502
		Si-OMg	1.584	1.589
		SiO-Mg	1.861	1.790
		Mg-OMg	1.943	1.894
		Mg_2_O–H	0.959	
	bond angles (deg)	O=Si-OMg	142.1	133.1
		O=Si-OSi	123.0	124.5
		Si–O–Mg	99.68	144.8
		Mg–O–Mg	114.7	
		Si–O–Si	138.5	
		Si_2_O-Mg_2_O-H	115.7	157.6
		SiO–Mg-H	152.7	131.4
structure L1 chiral dimer with molecular hydrogen (Mg_2_Si_2_O_6_–H_2_)	bond lengths (Å)	Mg–Si	2.742	3.105
		H-OSi_2_	--	2.189
		H–H	0.755	
		O=Si	1.509	1.510
		Si-OMg	1.580	1.579
		SiO-Mg	1.881	1.849
		Mg-OMg	1.805	1.791
	bond angles (deg)	O=Si-OMg	136.4	131.6
		O=Si-OSi	124.6	122.4
		Si–O–Mg	104.4	129.8
		Mg–O–Mg	113.5	
		Si–O–Si	141.1	
		H-OSi_2_–H	7.873	
		H-OMg_2_–H	11.22	

##### 3.1.1.1 Effect of Hydrogenation on the Enstatite Monomer

None of the hydrogenated monomers exhibit the 3D chirality required for enantioselectivity. Adding a neutral H to the enstatite monomer results in four stable structures (B1–B4 in Figure 1), where Structure B2 is the most stable. The structure retains its overall monomer shape, but its symmetry is reduced to *C_S_*, with the H sitting at a 117.1° angle from the silicon atom and double-bonded oxygen as the vertex.

The addition of a proton results in two structures with two-dimensional chirality with *C_S_* symmetry (Structures C1 and C2 in Figure 1). The overall shape is identical to that of structure B2, with minor deviations in bond angles and lengths. The H now sits at a slightly larger 124.2° angle.

Molecular hydrogen produces the least number of changes to the original structure and exhibits *C*_2*V*_ symmetry (D1 in Figure 1), and it is the only hydrogenated structure to preserve all of the symmetry elements of its parent molecule. The enstatite monomer retained the same overall shape as its parent molecule (Figure 1A) with the H_2_ coplanar with the ring and interacting with the Mg atom. The calculated bond length for H_2_ is reasonable compared with experimental values (0.74 Å). Any one of the H atoms sits much farther away from any of the enstatite monomer atoms compared to the single H and H^+^ structures. Another structure, D2 in Figure 1, was predicted to be a transition-state structure.

##### 3.1.1.2 Effect of Hydrogenation on the Enstatite Achiral Dimer [Structure E]

The bare achiral enstatite dimer (Structure E) is shown in Figure 2. The structure’s relatively low starting symmetry makes it easier to place it into *C*_1_ symmetry with the addition of one of the studied adsorbates (see Section 3.1.1.3). It continues to make use of three-coordinate Si atoms and is planar with *C*_2*V*_ symmetry.

Adding neutral H to the bare dimer produces a three-dimensional structure with the O connected to the two Mg atoms being lifted off the plane from the rest of the enstatite atoms (Structure F, Figure 2). The hydrogen occupies the space opposite to it, with the remaining enstatite atoms acting as the dividing place between the H and the O atoms. This produces a *C_S_* symmetry.

On the other hand, a proton to the achiral enstatite dimer results in a planar molecule (Structure G, Figure 2), where the proton sits in the middle of the ring. The O atom connected to the two Mg atoms reduces to the O atom connected to the two Si atoms. The angle created by the Mg–O–Mg portion of the ring is nearly 180°, producing a nearly linear component to the ring. The resulting structure has *C*_2*V*_ symmetry.

As with the monomer, the addition of molecular hydrogen (H_2_) provides the fewest changes to the unhydrogenated structure (Structure H, Figure 2). The original enstatite dimer structure remained largely unchanged. The H_2_ sits above the two dangling O atoms. The calculated interatomic distance between the two H atoms falls by 0.01 Å, indicating that the two O atoms might be somewhat compressing the two H atoms together. The enstatite components of the complex remain planar, and the complex has an overall *C_S_* symmetry.

While hydrogenation of the achiral enstatite dimer does not produce a chiral structure, large effects appear in the calculated frequency modes (Section 3.2.2.2).

##### 3.1.1.3 Effect of Hydrogenation on the Chiral Enstatite Dimer [Structure I]

The two-dimensional chiral conformer (Structure I, Figure 3) of the achiral enstatite dimer consists of the same atoms, but one of the O–Mg–O portions is linear, producing a two-dimensional chiral structure. The molecule is planar, so it still has *C_S_* symmetry. This is a somewhat strained geometry. However, it is a good candidate for trying to find a chiral structure with an adsorbed species as it is even lower in symmetry than structure E. As compared to its more symmetric isomer, the bond angle created by Mg–O–Mg falls from about 117 to 113°, and the angle created by Si–O–Si falls from 148° to about 141°. Nevertheless, the chiral dimer appears to be a stable–albeit higher energy–conformer of the achiral version with no imaginary frequencies predicted by the HSE06/aug-cc-pVQZ level of theory (see the next section).

Adding a neutral H to the bare chiral enstatite dimer produces several three-dimensional chiral molecules (Structures J1–J4, J6, and J7 in Figure 3). The first set of chiral structures, J1–J4, displays axial chirality, whereas structures J6 and J7 have stereogenic centers located at the silicon atoms. The three-dimensional chiral molecules introduce the possibility of optical activity (see Section 3.3.4).

**Figure 4:**
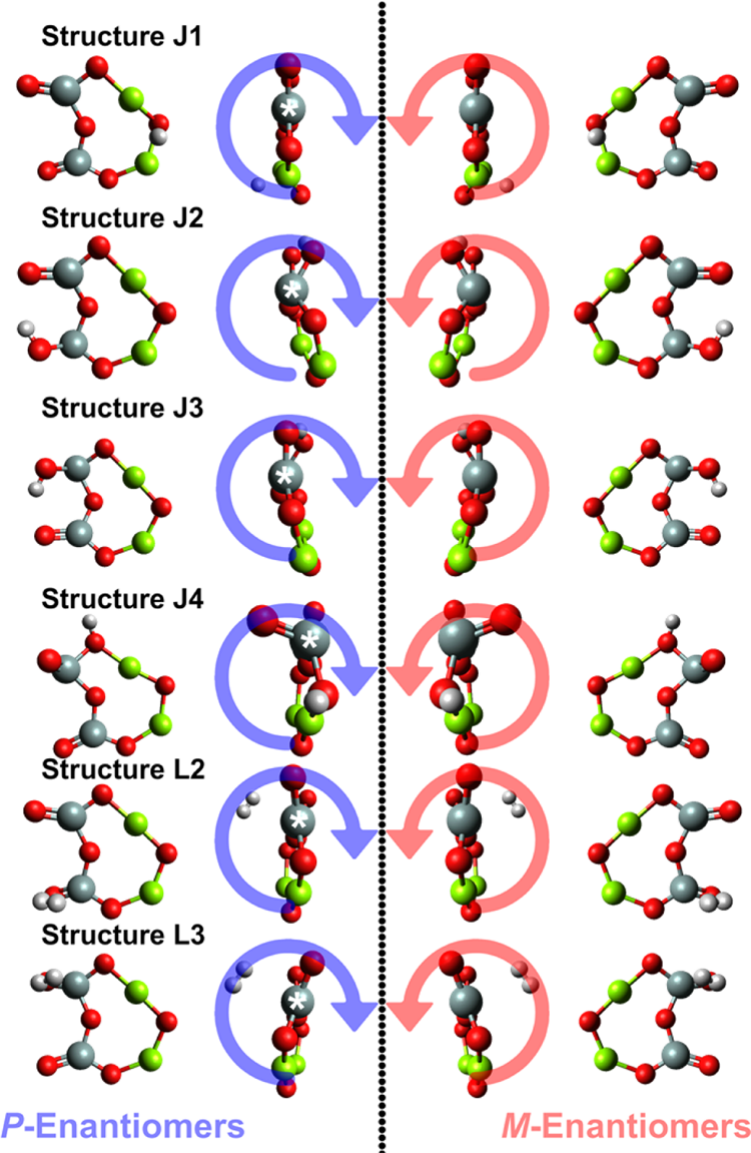
Three-dimensional axially chiral structures. The structure consists of the two-dimensional chiral conformer bonded with either a neutral hydrogen atom (J1–4) or molecular hydrogen (L2 and L3). The complexes are not planar, which produces a three-dimensional chirality that makes them candidates for symmetry breaking early in the enstatite forming process. There is no stereogenic center (see Figure 5). Rather, the chirality comes from the axial chirality around the axis produced by the line running through the two Si atoms. This produces a *P*- and *M*-enantiomer for the shown structures.

**Figure 5:**
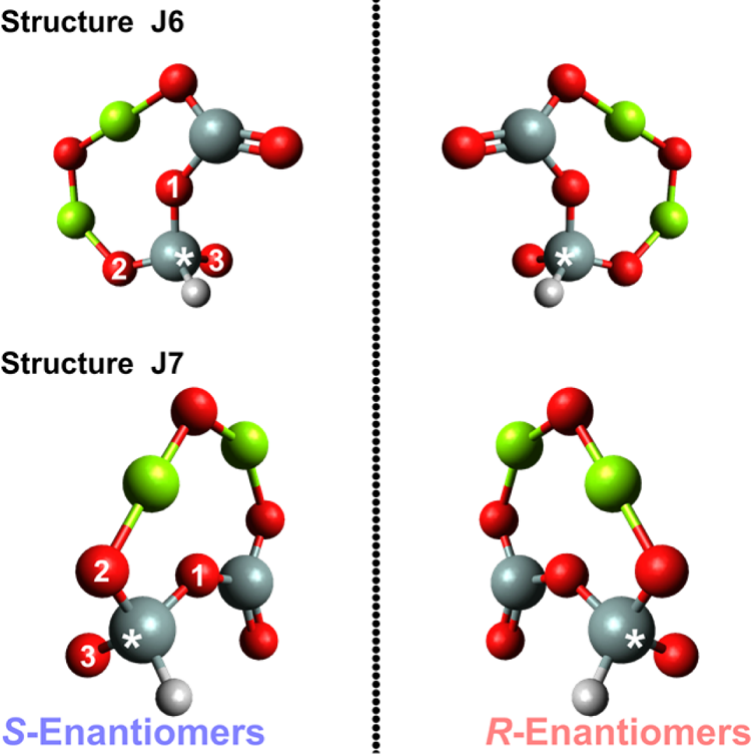
Chiral molecules with a stereogenic center with both enantiomers are shown for each molecule. Both involve a neutral hydrogen with the chiral dimer acting as the base silicate. The only difference is that the silicon atom is acting as the stereogenic center. These are marked with a star (*). The priority groups are numbered for the *S*-enantiomers.

Axial chirality is usually described utilizing *P*/*M* notation as opposed to the more familiar *S*/*R*, (+)/(−) or *l*/*d* notations.^87^ The hydrogenated nanosilicate structure fundamentally differs from other molecules utilizing *P/M* notation, such as biphenyls, allenes, alkylidenecycloalkanes, spiranes, helicenes, ansa compounds, and paracyclophanes, in that there are no functional groups that one can use to assign priority to determine the front-facing portion of the axis. However, one can use the example of the H_2_O_2_ conformer, a molecule described with *P/M* notation,^88^ and also use the fact that higher priorities are given to functional groups with higher mass.^89^ Using structure J1 as an example allows us to choose the side with the linear O–Mg–O bond as a higher priority, as more of the mass of the molecule rests toward that direction. Using this as the front-facing part of the axis, one then can apply the usual priority rules to go either clockwise or counterclockwise to assign the *P*- and *M*-enantiomer (Figure 4). Figure 5 shows both *R*-/*S*-enantiomers for structures J6 and J7, marking the stereogenic silicon atoms with a star.

The protonated chiral dimer (Structure K, Figure 3) is optimized (energy is minimized) when a proton bonds to the oxygen shared by the two Mg atoms. This structure has a two-dimensional chirality to it. The oxygen shared by the two Si atoms also interacts with one of the Mg atoms. This allows one to see the enstatite monomer (Structure A, Figure 1), within the molecule, very easily. Another striking feature of this molecule is that the proton lies outside the ring. In the hydrogenated 3D chiral dimer (Structure J, Figure 3), the role of the neutral H is analogous to that of the H atom in the achiral dimer (Structure F, Figure 2), causing the O atom to move out of the plane from the rest of the molecule. However, in the protonated chiral enstatite, the charged proton does not take the analogous role of resting in the ring of the enstatite dimer but rather sits outside the ring. As with the bare chiral dimer, the molecule is planar, so it retains some symmetry, giving it *C_S_* symmetry.

As for all of the other enstatite nanostructures with molecular hydrogen, Structure L1 (Figure 3) exhibits the fewest changes when compared to the parent enstatite structure. It is the lowest energy structure among the chiral dimers with H_2_. In structure L1, the H_2_ lies in a plane with the enstatite dimer positioned closest to the Mg atom that is not part of the linear O–Mg–O bond. This also differentiates it from structure H, where the H_2_ sits above the dangling O atoms, and makes it more akin to the H_2_-monomer, where the H_2_ is positioned closest to the Mg atom. The entire complex remains planar, which gives it *C_S_* symmetry.

Hydrogenation in the case of the 2D chiral dimer produced one 3D chiral molecule with neutral H and preserved the 2D chirality of the original bare silicate in the case of protonation and adsorption with H_2_. As with the achiral dimer, the variation in structure resulted in a diverse range of frequency modes (Section 3.1.2.2).

#### 3.1.2 Frequency Calculations

**Figure 6:**
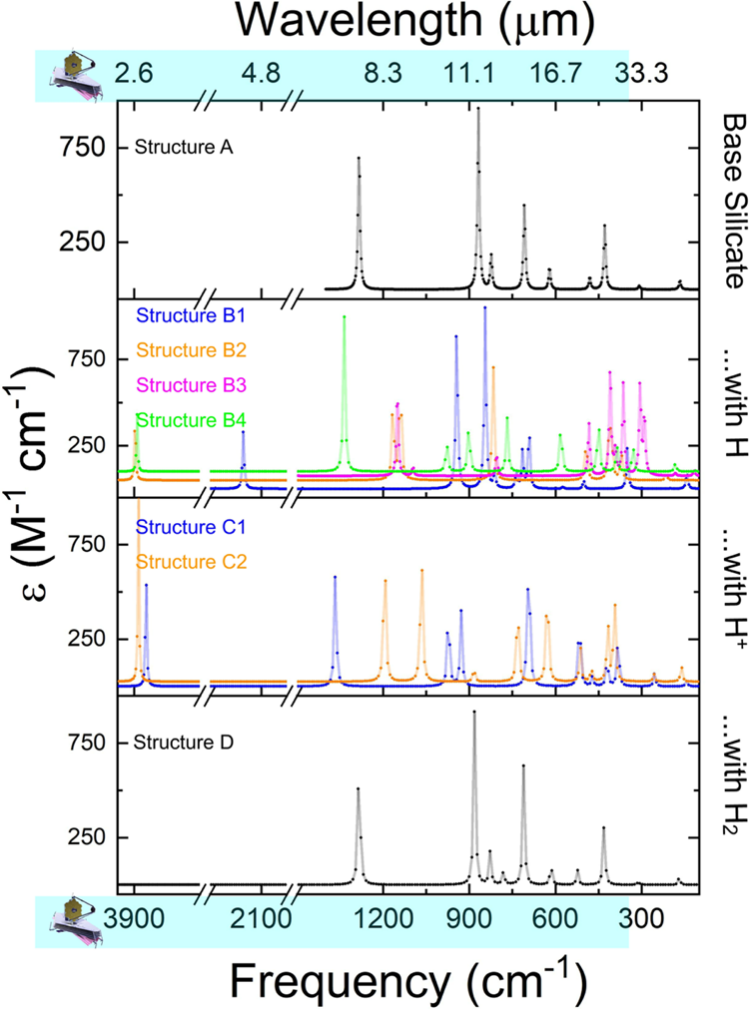
Unscaled harmonic frequencies of the various monomer structures. The monomer (Structure A) frequencies match well with previous results (Valencia et al., 2020). Hydrogenating the structures results in a shift to largely higher frequencies (smaller wavelengths). Most of these still fall within the range that can be detected by the JWST. Peaks falling below 10 μm might be difficult to differentiate from PAHs.

**Figure 7:**
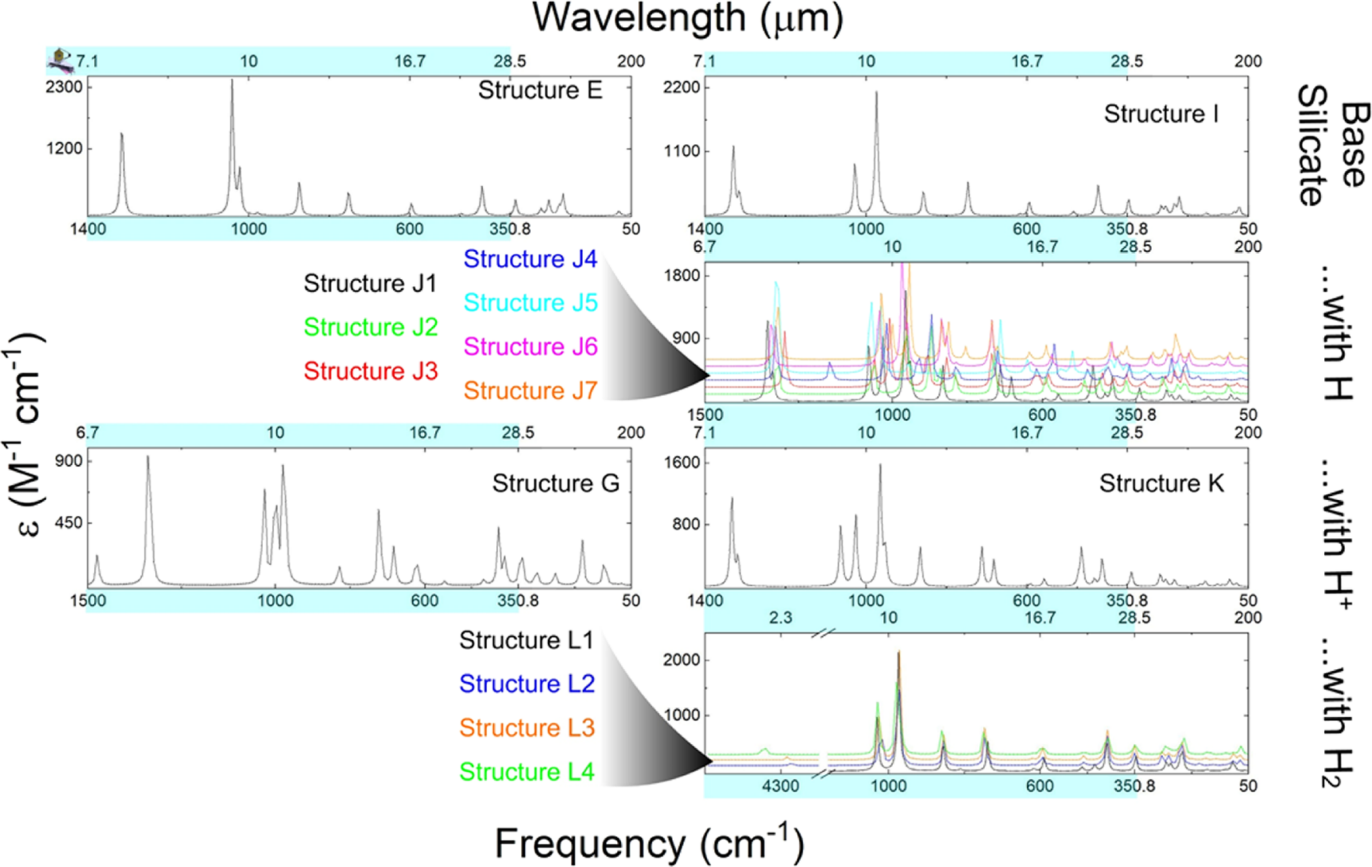
Unscaled harmonic frequencies of the various achiral (left-hand side) and chiral (right-hand side) dimer structures (from top to bottom, Structures E and I; the J structures on the chiral side; Structures G and K; and the L structures on the chiral side). All of the major modes fall within the range that can be detected by the JWST. The most dominant peaks appear at higher frequencies than what is normally expected for bulk silicate material for most of the structures. Peaks falling below 10 μm might be difficult to differentiate from PAHs. There are no spectra shown for the achiral conformer with neutral hydrogen and the achiral conformer with molecular hydrogen because they represented transition-state structures.

The frequency calculations revealed that the H- and H_2_- enstatite achiral dimers (structures F and H from Figure 2, respectively) were transition-state structures, as each of those had an imaginary frequency. The same can be said for monomeric Structure D2 (Figure 1) from the monomers. Otherwise, the rest of the structures represent stable conformers of the studied molecules or complexes. A notable observation is that most of the IR modes predicted by the calculations can be observed with JWST, as shown in Figures 6 and 7. This is consistent with other studies of pyroxene nanosilicates,^21,29,31^ though these calculations predict for some peaks to occur at even lower wavelengths that are most commonly associated with organics like PAHs.

##### 3.1.2.1 Monomer Structures—Dominant Vibrational Modes

The DFT frequency calculations using the HSE06/aug-cc-pVQZ level of theory matched the CCSD(T)-F12/cc-pVTZ-F12 spectral results of Valencia et al.^21^ well, at a fraction of the computational cost compared to higher levels of theory (i.e., CCSD). For the bare enstatite monomer (Structure A), the three most intense modes are ω_2_ (868.2 cm^–1^, 11.51 μm), ω_1_ (1284.3 cm^–1^, 7.78 μm), and ω_4_ (708.1 cm^–1^, 14.12 μm). Once the HI is added, producing the most energetically favorable conformer (Structure B2), the most intense modes are ω_3_ (1139.8 cm^–1^, 8.77 μm), ω_5_ (816.7 cm^–1^, 12.24 μm), and ω_2_ (1165.2 cm^–1^, 8.58 μm). Protonating the bare monomer, the most stable structure (C2) gives ω_1_ (3872.6 cm^–1^, 2.58 μm), ω_3_ (1066.6 cm^–1^, 9.37 μm), and ω_2_ (1195.0 cm^–1^, 8.36 μm) as the three strongest modes. The first mode is exceptionally bright, surpassing 300 km/mol. Here, the latter two IR features are further blue-shifted, appearing in the region frequently attributed to polycyclic aromatic hydrocarbons (PAHs).^90,91^ The enstatite monomeric structure with H_2_ (Structure D1) has its brightest features at modes ω_3_ (881.8 cm^–1^, 11.34 μm), ω_2_ (1284.0 cm^–1^, 7.78 μm), and ω_6_ (710.7 cm^–1^, 14.07 μm). The brightest two are above 200 km/mol, whereas the third comes close at 183 km/mol. The second IR feature is slightly blue-shifted from where the most intense amorphous silicate peaks are thought to occur in PPDs and the ISM, which is to say it falls below 10 μm.^16,31,92,93^

The predicted IR spectra of the bare monomer were affected by the hydrogenation type of the structure to a large degree, with only the bare monomer and monomer with H_2_ showing any significant overlap. Otherwise, hydrogenation of the monomer had the effect of producing vibrational features at lower wavelengths. As stated, the bare monomer showed good agreement with the harmonic calculations from Valencia et al.,^21^ where the authors’ harmonic and anharmonic calculations for the monomer showed that ω_1_ and *v*_1_, respectively, fall below peaks commonly associated with silicates.^16,31,92,93^ The hydrogenated versions of the monomers strengthen this effect (Figure 6, Table 4) with several modes falling well within the range of wavelengths commonly associated with the C–H in-plane bending and C–C bending of PAHs^94^ and mixed organic aromatic/aliphatic nanoparticles, abbreviated as MOANs.^95,96^

**Table 4:** Monomer Frequency Calculations at HSE06/aug-cc-pVQZ for Selected Structures from Figure 1

structure	A		B2		C2		D1	
	monomer (MgSiO_3_)		monomer with neutral hydrogen (MgSiO_3_–H)		monomer with proton (MgSiO_3_–H^+^)		monomer with molecular hydrogen (MgSiO_3_–H_2_)	
mode	cm^–1^	km/mol	cm^–1^	km/mol	cm^–1^	km/mol	cm^–1^	km/mol
ω_1_	1284.3	213	3892.6	142	3872.6	304	4245.6	69
ω_2_	868.2	278	1165.2	156	1195.0	239	1284	232
ω_3_	832.5	52	1139.8	204	1066.6	243	881.8	265
ω_4_	708.1	129	871.2	2	883.7	24	827.1	52
ω_5_	620.3	34	816.7	194	731.6	148	780.5	24
ω_6_	480.2	19	492.4	86	628.2	190	710.7	183
ω_7_	428.2	98	411.6	142	512.8	52	615.3	36
ω_8_	307.9	5	400.5	33	474.9	22	522.9	23
ω_9_	166.2	14	387.0	1	418.0	101	433.1	4
ω_10_			370.1	50	394.0	142	430.5	95
ω_11_			213.7	11	257.1	12	310.7	5
ω_12_			129.2	0	161.4	24	172.4	0
ω_13_							168.8	12
ω_14_							59.5	0
ω_15_							36.8	0

Ultimately, more accurate computational methods or even experimental data will be required to obtain a better picture of the actual IR spectra of these hydrogenated enstatite nanosilicates. However, it is unlikely that the modes fall in frequencies far outside the values calculated by HSE06/aug-cc-pVQZ. This suggests that a fraction of the infrared wavelengths commonly associated with PAHs might actually be the result of hydrogenated nanosilicates, enstatite, or otherwise. This makes sense as the behavior of the Si–H bonds mimics the behavior of the C–H bonds. The mid-infrared is a region of the electromagnetic spectrum that is sensitive to chemical functional groups, so assigning specific molecules to spectral features is nontrivial once one escapes the relative simplicity and controlled environment of the laboratory.

##### 3.1.2.2 Dimer Structures

The frequencies of the various achiral enstatite dimers are summarized in Table 5. Beginning with the bare achiral dimer (Structure E), the three brightest IR features are ω_3_ (699.0 cm^–1^, 14.3 μm), ω_1_ (1315.5 cm^–1^, 7.6 μm), and ω_4_ (1023.0 cm^–1^, 9.77 μm). Hydrogenating the achiral enstatite dimer with HI (Structure F) and with H_2_ (Structure H) create transition-state molecular structures due to imaginary frequencies at (−42.5 cm^–1^) and (−168.8 cm^–1^), respectively. The achiral enstatite dimer with the H^+^ (Structure G), however, shifts the brightest peaks down in wavelength by almost a factor of 2 for ω_1_ and ω_3_, where ω_1_ (3064.8 cm^–1^, 3.26 μm), ω_7_ (976.7 cm^–1^, 10.23 μm), and ω_3_ (1337.2 cm^–1^, 7.47 μm).

**Table 5:** Achiral Dimer Frequency Calculations at HSE06/aug-cc-pVQZ for Figure 2 (Structures E–H)

structure	E		F		G		H	
	achiral dimer (Mg_2_Si_2_O_6_)		achiral dimer with neutral hydrogen (Mg_2_Si_2_O_6_–H)		achiral dimer with proton (Mg_2_Si_2_O_6_–H^+^)		achiral dimer with molecular hydrogen (Mg_2_Si_2_O_6_–H_2_)	
mode	cm^–1^	km/mol	cm^–1^	km/mol	cm^–1^	km/mol	cm^–1^	km/mol
ω_1_	1315.5	456	1321.6	444	3064.8	906	4374.1	4
ω_2_	1310.4	63	1314	37	1474.3	74	1314.1	459
ω_3_	1041.5	699	1023.3	207	1337.2	396	1309	60
ω_4_	1023	228	1022.2	928	1329.5	44	1044.8	681
ω_5_	977.9	11	1005.8	66	1030.1	247	1024.3	238
ω_6_	874.3	180	967.4	3	999.7	274	979.2	12
ω_7_	752.2	131	926.8	79	976.7	402	874.6	181
ω_8_	604	0	723.5	113	830	47	752	128
ω_9_	596	64	710.2	129	722	198	603.3	0
ω_10_	472.8	10	586.5	4	682.9	86	595.7	63
ω_11_	429	14	553.5	23	623.5	74	471.5	10
ω_12_	420.1	153	467	7	548.4	8	428.6	16
ω_13_	378.9	0	459	116	442.7	11	419.2	157
ω_14_	337.4	80	428.4	46	403.7	118	379.8	0
ω_15_	274	33	411.9	114	386.6	59	339.1	63
ω_16_	254.9	78	376.7	0	364.4	0	325.2	18
ω_17_	229.3	43	337.3	68	343.4	97	274.9	31
ω_18_	219.2	107	270	32	303.2	40	255.3	79
ω_19_	140.7	0	251.4	26	260	3	229.2	43
ω_20_	106.1	1	229.7	41	252.8	24	218.8	106
ω_21_	81.2	17	163	23	181.1	101	148.7	0
ω_22_	79.2	7	142.4	2	127.6	0	135.6	0
ω_23_	44	44	118.5	8	121.8	8	115.4	1
ω_24_	19.7	0	85.1	14	120.7	59	103.5	4
ω_25_			78.9	10	95.6	1	85.9	13
ω_26_			20.7	1	77.5	3	78.2	9
ω_27_			–42.5	39	22.4	0	46.8	44
ω_28_							32.5	1
ω_29_							10.9	0
ω_30_							–168.8	1

Table 6 shows the structure variations of the chiral enstatite dimer. The bare chiral dimer (Structure I) has its most intense IR peaks at ω _4_ (974.7 cm^–1^, 10.25 μm), ω_1_ (1330.5 cm^–1^, 7.51 μm), and ω_3_ (1028.0 cm^–1^, 9.72 μm). Hydrogenating the chiral enstatite with neutral H (Figures 3–5) produces several three-dimensional chiral nanosilicate molecules, but the most stable one is the achiral structure J5. Its most intense vibrational modes are at ω_4_ (1058.8 cm^–1^, 9.44 μm), ω_2_ (1313.2 cm^–1^, 7.61 μm), and ω_5_ (1033.9 cm^–1^, 9.67 μm). The protonated structure (Structure K) had its three most intense modes at ω_6_ (919.5 cm^–1^, 10.87 μm), ω_4_ (1056.4 cm^–1^, 9.46 μm), and ω_5_ (738.7 cm^–1^, 13.53 μm). Finally, we find that the most energetically stable chiral dimer with H_2_ (Structure L1) has its most intense peaks at ω_5_ (974.4 cm^–1^, 10.27 μm), ω_2_ (1330.6 cm^–1^, 7.52 μm), and ω_4_ (1030.9 cm^–1^, 9.70 μm).

**Table 6:** Chiral Dimer Frequency Calculations at HSE06/aug-cc-pVQZ for Selected Structures from Figure 3

structure	I		J5		K		L1	
	chiral dimer (Mg_2_Si_2_O_6_)		chiral dimer with neutral hydrogen (Mg_2_Si_2_O_6_–H)		chiral dimer with proton (Mg_2_Si_2_O_6_–H^+^)		chiral dimer with molecular hydrogen (Mg_2_Si_2_O_6_–H_2_)	
mode	cm^–1^	km/mol	cm^–1^	km/mol	cm^–1^	km/mol	cm^–1^	km/mol
ω_1_	1330.5	355	3961.5	85	3882.3	147	4258.7	36
ω_2_	1315.2	109	1313.2	341	1358.9	285	1330.6	357
ω_3_	1028	263	1304.6	300	1342	104	1314.8	122
ω_4_	974.7	620	1058.8	425	1056.4	294	1030.9	287
ω_5_	959	26	1033.9	332	946.8	28	974.4	622
ω_6_	857.8	130	956.6	62	919.5	656	959.3	20
ω_7_	747.4	166	726.2	34	903.9	24	856.3	142
ω_8_	617.4	7	711.5	224	720.5	251	810.6	12
ω_9_	594.8	72	642.1	71	651.9	112	738.7	156
ω_10_	484.9	21	549.0	26	578.9	15	617.1	12
ω_11_	430.9	15	520.8	94	558.8	1	589.6	72
ω_12_	423.4	150	435.9	17	494	95	489.6	21
ω_13_	375.8	0	420.2	100	435.5	210	456.2	35
ω_14_	348.2	83	398.0	82	421.2	4	429.1	11
ω_15_	266.7	44	391.6	6	415.6	85	424.2	149
ω_16_	255.7	42	374.7	40	360.4	1	376.9	0
ω_17_	234.1	53	372.7	0	355.9	98	348	78
ω_18_	221.8	90	326.2	13	287.3	35	302.4	0
ω_19_	154	11	244.6	14	239.7	33	267.2	45
ω_20_	115	5	240.3	46	223.8	8	255.5	34
ω_21_	109	3	212.4	10	194.6	25	230.2	56
ω_22_	86.1	15	201.2	2	192.2	19	224.1	83
ω_23_	73.3	44	132.2	3	146.5	21	158.5	8
ω_24_	17.5	1	104.6	2	97.4	4	117	3
ω_25_			95.1	4	75.5	17	109.4	5
ω_26_			73.1	11	73.5	1	92.8	43
ω_27_			42.2	4	26.7	0	86.7	17
ω_28_							60.8	0
ω_29_							57.9	4
ω_30_							17.8	1

Clearly, hydrogenating the dimer structures has a large effect on the IR spectra (Figures 6 and 7, Tables 5 and 6), where the IR modes are significantly blue-shifted. Similar to the monomers, these vibrational modes are significantly lower in wavelength than those commonly associated with silicates, which have major vibrational frequencies at about 10 μm or higher.^16,31,92,93,97,98^ It is unclear whether this would continue to be the case if more monomer subunits were added to the structure. However, the structure would ultimately start to behave like a bulk amorphous or crystalline enstatite if enough subunits are added. Larger hydrogenated pyroxene nanosilicates studied by Goumans and Bromley^28^ already show behavior more similar to the bulk (absorbance ≥10 μm), crystalline species, though they too noted a blue-shifting of the peaks. Their structures consisted of the stoichiometric equivalent of four monomers (i.e., tetramers).

##### 3.1.2.3 Astronomical Implications of the Frequency Calculations

The wavelength of the calculated nanosilicate vibrational modes, especially the location of the strongest emission lines, suggests that they have some overlap with the range associated with PAHs and could contribute to the IR spectrum alongside hydrocarbons. Valencia et al.^21^ noted that bulk enstatite has been found in NGC 6302 (Butterfly Nebula), with Molster et al.^17^ attributing many features below 10 μm to PAHs. However, as shown by our calculations, many of these emission lines, along with many of the unidentified IR features, are also consistent with hydrogenated enstatite nanosilicate monomers and dimers. In particular, the 3.29 μm feature attributed to PAH C–H stretching is consistent with the protonated achiral enstatite dimer (Structure G), and the protonated monomer (Structure C) also has a mode near 2.6 μm, which is near bright and unassigned spectral features in the Infrared Space Observatory (ISO) data.^17^ Furthermore, almost all of the nanosilicates have predicted vibrations near 7.6 μm, currently fully attributed to PAH C–C stretching in the ISO data.^17^ We find that the bare achiral dimer (Structure E) has an emission at 7.6 μm with several of the other dimer structures sitting right below it (7.4–7.5 μm for structures I, J1–7, and K). The bare monomer (Structure A) and the monomer with molecular hydrogen (Structure D1) also have predicted spectral features of 7.8 μm.

Also of note in the ISO data is a bright feature near 11.01 μm assigned as “PAH+artifact?”,^17^ which is consistent with absorption bands present in several of the studied nanosilicate structures. These include a monomer with neutral H (Structure B1), the chiral dimer (Structure I), several of the chiral dimers with neutral hydrogen (all J structures except for J4), all of the chiral dimers with molecular hydrogen (structures L1–L4), and the achiral dimer with H^+^ (Structure G), which all fall within 0.7 μm of the observed feature. One can find matches for the three most intense spectral features of each hydrogenated nanosilicate in the ISO data for NGC 6302,^17^ except for the protonated achiral dimer (Structure G), which has the strongest peak at ∼10 μm. However, this Si–O feature falls within 0.5 μm of the 11.01 μm observed feature, so it cannot be completely disregarded.

Two outstanding problems in astrochemistry are the unidentified infrared emission bands (UIBs) and diffuse interstellar bands (DIBs),^90^ with the latter having adsorption features seen in the ultraviolet (UV), visible, and IR wavelengths. While C_60_^+^ has been identified as contributing to several DIBs,^99^ there are hundreds of DIBs that remain unidentified.^90,100^ Several DIB surveys have been conducted, with most in the visible range but some in the infrared range;^100,101^ none of our nanosilicate structures (bare or hydrogenated) match DIBs in this part of the spectrum.

While the putative sources are PAHs or other carbonaceous material for DIBs and UIBs,^102,103^ the bright features corresponding to wavelengths below 10 μm for the studied hydrogenated nanosilicates means that other sources cannot be disregarded. This is not to say that the studied hydrogenated nanosilicates or similar structures would be solely responsible for these features, but rather that they could contribute to the signals alongside the putative hydrocarbons. Furthermore, energy calculations show that the studied hydrogenated nanosilicates are relatively stable structures (Section 3.3.3). However, due to the base silicates for the dimers being high-energy metastable isomers, it is unlikely that they would actually be found in great quantities in the ISM. Nevertheless, the lower energy isomers studied by Valencia et al.^21^ could exhibit similar spectral features if hydrogenated due to similar bending and stretching behaviors between the various forms of hydrogen and the atoms they are adsorbed to Si, O, Mg, and such hydrogenated structures should be a target of future calculations to confirm this.

Finding candidates for UIBs is more likely due to their sharp, distinct features compared to DIBs. In particular, spectral features at 3.3, 6.2, 7.7, 8.6, and 11.3 μm are especially interesting.^103^ The 3.3 μm band is usually attributed to an aromatic C–H stretch, which is close to that of studied PAHs, though not an exact match.^103^ However, as our calculations reveal, the feature is also consistent with the achiral dimer with a proton (Structure G). The hydrogenated nanosilicates even make good candidates for other features. As mentioned, most of the predicted molecules have a peak at near 7.7 μm. All of the dimer structures have bright features around 10–10.5 μm, which is also consistent with bulk silicates.^97,98^

The monomer structures remain consistent with a number of structures having features near 11.3 μm (structures A and D1), commonly assigned to C–H out of plane bending in PAHs, and near 14–14.5 μm (all monomer structures). Lastly, the 3.3 and 11.3 μm features are correlated, which has previously led to the suggestion that the two have a common origin^104^ and were originally interpreted as being caused by silicates or other minerals.^104,105^ Carbonate was ruled out due to a lack of 7.0 μm feature,^103^ but, as shown, the predicted frequencies of hydrogenated nanosilicates share some of the same modes as PAHs and can help explain unidentified peaks. Though most of the UIBs are probably the result of C-containing molecules,^103^ it is not unreasonable to think that structures intermediate of bulk crystalline silicates and individual atoms are present in the ISM and may contribute to the infrared features observed. Unlike the dimer species, the monomer is much more likely to be found in the ISM as it is a stable structure.

We note that the current study did not aim to address the identity of unassigned spectral features in the 1–10 μm range of astronomical observations. Valencia et al. (2021), Bromley et al. (2014), Kerkeni et al. (2017), Oueslati et al. (2015), Rimola and Bromley (2021), Goumans and Bromley (2011), and Escatllar et al. (2019) have identified other nanosilicates with either pyroxene or forsterite stoichiometries. In many cases, these were much larger structures and true nanosilicates (being at least a nanometer in length in one dimension). Goumans and Bromley (2011), Oueslati et al. (2015), and Kerkeni et al. (2017) studied the hydrogenation of enstatite nanosilicate computationally with some of these larger clusters and, while blue-shifting of the peaks was observed, did not find a large shift of the most intense features to 2–10 μm. This suggests that the size of the molecule matters for the shift to occur. Furthermore, the hydrogenation of most other nanosilicate structures has not been studied. Valencia et al. (2021) presented four other isomers with enstatite stoichiometry, three monomers with forsterite stoichiometry, and eight dimers with forsterite stoichiometry. It is possible that hydrogenated versions of these other Mg-containing nanosilicates show similar vibrational shifts and have their infrared spectra intermixed in regions commonly associated with PAHs. Hydrogenated silicon oxides and other stoichiometries based on common astronomical minerals could potentially show similar effects. It is further possible that these routes could explain some DIB or UIB bands even better. Therefore, they should be considered as important targets for both computational and laboratory studies. The latter is especially important for assigning spectral features from astronomical observations to nanoparticles of nanosilicate molecules.

### 3.2 HSE06/aug-cc-pVQZ Energy Calculations

The single-point energies of structures studied by Valencia et al.^21^ were recalculated with DFT using the HSE06/aug-cc-pVQZ level of theory (Table S4). Single-point energy calculations were also conducted on the optimized structures shown in Figure 1, and the results are summarized in Table S5. Values described in these tables provide a way to calculate the total electronic energy of a system at a fixed geometry, which can be used to compare the relative stabilities of different structures. These can be used to (1) calculate the adsorption energies (Table 7), (2) show the relative energies between analogous achiral and chiral pairs (Table 8), and (3) obtain the synthesized molecule rotational constants (Table 9).

**Table 7:** Adsorption Energies for Structures Described in Figures 1–3

structure	complex	energy (eV)	energy (kcal mol^–1^)
B1	MgSiO_3_ + H	1.25	28.77
B2		3.59	82.5
B3		1.17	26.9
B4		3.17	73.2
C1	MgSiO_3_ + H^+^	9.48	218.68
C2		10.6	243.74
D1	MgSiO_3_ + H_2_	0.30	6.90
D2		0.058	5.55
F	Achiral Mg_2_Si_2_O_6_ + H	0.67	15.43
G	Achiral Mg_2_Si_2_O_6_ + H^+^	8.81	203.09
H	Achiral Mg_2_Si_2_O_6_ + H_2_	0.03	0.71
J1	Chiral Mg_2_Si_2_O_6_ + H	0.70	16.15
J2		2.87	66.16
J3		2.85	65.80
J4		1.59	36.62
J5		3.64	83.93
J6		1.56	35.89
J7		1.53	35.30
K	Chiral Mg_2_Si_2_O_6_ + H^+^	9.76	224.96
L1	Chiral Mg_2_Si_2_O_6_ + H_2_	0.25	5.68
L2		0.070	1.61
L3		0.069	1.57
L4		–27.2	–626

**Table 8:** Relative Energies of Dimer Conformers (Structures Shown in Figures 2 and 3)

	structure	complex	HSE06/aug-cc-pVQZ (a.u.)	relative energies (kcal mol^–1^)
neutral H dimers	*F*	Achiral Mg_2_Si_2_O_6_ + H	–1430.853349	0.71
	J1	Chiral Mg_2_Si_2_O_6_ + H	–1430.854478	68.48
	J2		–1430.934184	67.77
	J3		–1430.933601	17.76
	J4		–1430.887099	18.12
	J5		–1430.962484	0.00
	J6		–1430.885927	48.04
	J7		–1430.884995	48.62
protonated dimers	G	Achiral Mg_2_Si_2_O_6_ + H^+^	–1430.65096	21.86
	K	Chiral Mg_2_Si_2_O_6_ + H^+^	–1430.685802	0.00
dimers with molecular hydrogen	*H*	Achiral Mg_2_Si_2_O_6_ + H_2_	–1431.497589	4.96
	L1	Chiral Mg_2_Si_2_O_6_ + H_2_	–1431.505497	0.00
	L2		–1431.499008	4.07
	L3		–1431.498952	4.11
	L4		–1430.498125	632.13

**Table 9:** Rotational Constants (Structures Described in Figures 1–3)

	MgSiO_3_				Achiral Mg_2_Si_2_O_6_				Chiral Mg_2_Si_2_O_6_			
with...	--	H	H^+^	H_2_	--	H	H^+^	H_2_	--	H	H^+^	H_2_
structure	A	B2	C2	D1	E	F	G	H	I	J5	K	L1
point group	*C_2v_*	*C_s_*	*C_s_*	*C_2v_*	*C_2v_*	*C_s_*	*C_2v_*	*C_s_*	*C_s_*	*C_s_*	*C_s_*	*C_s_*
dipole (D)	12.3	1.92	6.92	13.5	12.3	*12*.*6*	23.5	*12*.*3*	11.5	10.8	21.8	12.3
A_e_ (MHz)	10009.4	9434.1	9859.6	9958.4	882.9	*901*.*1*	927.8	*863*.*3*	1037.2	1239.6	1198.5	1048.8
B_e_ (MHz)	3027.7	2744.5	2877.1	2561.6	865.9	*868*.*1*	804.6	*821*.*7*	797.9	635.9	723.5	734.8
C_e_ (MHz)	2324.6	2126.0	2227.2	2037.5	437.1	*448*.*3*	430.9	*429*.*1*	451.0	420.3	439.5	432.1

#### 3.2.1 Adsorption Energies

Adsorption energies (*E*_ads_) for the hydrogen species to enstatite nanosilicate were calculated from the data presented in Table S5 according to the equation *E*_ads_ = (*E*_enstatite_ + *E*_hydrogen_) – *E*_total_,^106^ where *E*_total_ is the total energy of the system (enstatite and hydrogen complex), *E*_enstatite_ is the total energy of the optimized enstatite monomer or dimer, and *E*_hydrogen_ is the total energy of either the H, H^+^, or H_2_. The adsorption energies were highest for enstatite nanosilicate species with the addition of a proton (∼9–11 eV). This suggests that protonated enstatite nanosilicates are very stable complexes. Adsorption of neutral hydrogen into the dimers varied greatly. The range for the monomers was ∼1.25 to ∼3.60 eV. For the dimers, Structures F and J1 both show relatively weak adsorption (∼0.67–0.70 eV) in contrast to Structure J5, which had an adsorption value of ∼3.6 eV. The weakest bonds occurred between the nanosilicates and the molecular hydrogen, with about 300 meV being predicted by the DFT calculations for the strongest bonds (Structures D1 and L1).

Sil et al.^107^ studied neutral H and H_2_ adsorption onto nanosilicate grains at the MP2/aug-cc-pVDZ level of DFT theory. They calculated a small adsorption energy of 0.05 eV for neutral H and about 0.1 eV for H_2_ on a silica surface. While our values for H_2_ on enstatite dimers and monomers are consistent, the energy required to bond neutral H is quite different. The neutral H adsorption energies for most of our complexes represent adsorption energies stronger than physisorption.^108^ The presence of a Mg atom provides additional stabilization of the interaction between neutral H and the nanosilicates. This is an interesting effect if it carries down to even larger grain-sized versions of enstatite since a certain fraction of any astrophysical surface will be covered by other molecular species (e.g., H_2_O); these additional species need to be accounted for to completely understand the interaction of gas-phase molecules adsorbing or interacting with a surface.^109^ The mineral cation is known to affect the adsorption of various chemical species in zeolites,^110,111^ so differences in adsorption energy with and without Mg are not surprising.

Oueslati et al.^26^ studied the adsorption and reactivity of neutral hydrogen (HI) with nanosilicates containing both forsterite and enstatite stoichiometries using DFT at the MPWB1*K*/6-31G(d) level of theory for Mg and Si atoms and using MPWB1*K*/6-31+G(d,p) for O and H atoms. They found that the enstatite structure had two physisorption sites, where the H adsorption energies ranged from 0.72 to 2.07 eV, more consistent with our results, further indicating the role that Mg may play in stabilizing the surface bond with neutral H. Surface reactions of H are known to be important in the formation of H_2_.^25,28,39−42^ However, the local surface chemistry could have profound effects on the formation rate of H_2_ if HI adsorption is more energetically favorable on certain surfaces.

Table 8 shows the relative energies between analogous chiral pairs, where the achiral form is compared with its chiral counterpart. The transition-state structures are in italics. As expected, the achiral dimer structure with neutral H (Structure F) is higher in energy compared to its chiral form (Structure J1). While a higher degree of molecular symmetry usually leads to lower energy, the open-shell configuration of these complexes means that the odd electron must be placed on one side of the molecule, which influences the shape and energetics of the resulting molecular orbitals. For the open-shell structures, an asymmetrical lower energy structure makes sense, as the unpaired electron can only be placed in an orbital on one side of the structure. However, the achiral dimer with a proton and with H_2_ was also higher in energy compared to the chiral dimer with the proton. The explanation here is most likely due to the placement of the proton in each structure. The protonated achiral enstatite (Structure G) has the proton placed within the ring itself, producing a more strained structure, as described in section 3.1. On the other hand, in the protonated chiral enstatite (Structure K), the proton lies outside of the ring, and the enstatite component is more similar in shape to its bare parent (Structure I).

#### 3.2.2 Rotational Constants

##### 3.2.2.1 Monomers

The constants that describe the rotational properties of the enstatite nanosilicates (Table 9) that were calculated are limited but show that the molecules’ properties are favorable for detection in all cases. Valencia et al.^21^ used a higher level of theory, namely, CCSD(T)-F12/cc-pVTZ-F12, for calculating the rotational constants of the monomer with three isotopes of Mg (^24^Mg, ^25^Mg, ^26^Mg); the reader should refer to those calculations and constants for a more accurate simulation of the unhydrogenated-enstatite rotational spectrum. It is worth noting that the hydrogenated versions of the monomer all have lower rotational constants than the bare monomer, providing them with a slight disadvantage in detectability. However, while the dipole moment for the monomer is already quite high (>12 D), the predicted values for one protonated monomer (Structure C1) and the monomer with H_2_ (structure D1) are even larger (>13 D). Only the monomer with neutral H (Figure 1B) fares relatively poorly in all of the physical parameters necessary for detection when compared to the bare monomer, but it is still easily detectable if present.

##### 3.2.2.2 Dimers

While the dipole moments for all structures are relatively high (>9 D in all instances), in the case of the dimers, all of the dipole moments are >12 D, with the protonated forms having dipole moments exceeding 21 D. The achiral dimer (bare and hydrogenated) shows greater dipole moments than its chiral counterpart. In addition, all of the rotational constants are large for all eight enstatite dimers. For the two dimer conformers, the chiral dimer has slightly larger rotational constants within each type of hydrogenation category. Only the chiral dimers with neutral H and H_2_ (Structures J1–J6 and L1–L4) are likely to be present in interstellar space, as the other calculated molecules form transition-state species.

Of course, the detectability of a molecule also depends on other factors, such as the specific observational technique and the sensitivity of the detection equipment; however, all structures studied have the physical properties for relatively easy detection if they exist in protoplanetary disks or the ISM.

### 3.3 TD-DFT Calculations

#### 3.3.1 UV–vis Electronic Spectra

**Figure 8:**
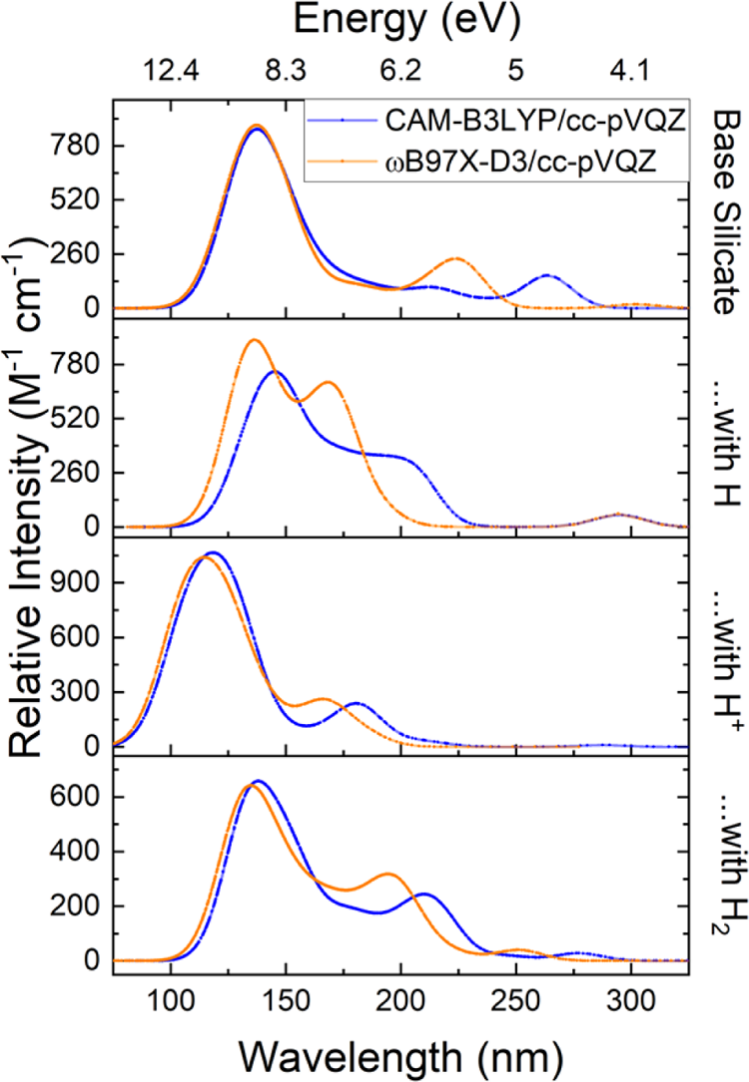
Predicted UV–vis spectra of the various monomer structures (from top to bottom, structures A, B2, C2, and D1) using the CAM-B3LYP/cc-pVQZ and ωB97X-D3/cc-pVQZ levels of theory. The spectra were created by using Avogadro with 60 transition states with a Gaussian line shape and a peak width of 24 nm.

**Figure 9:**
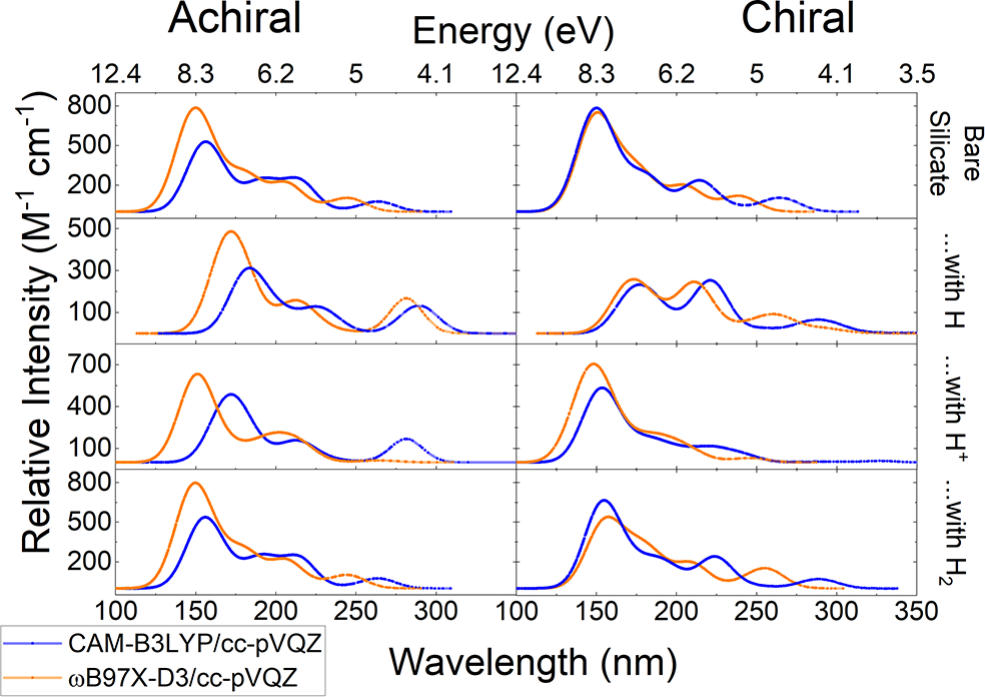
Predicted UV–vis spectra of both the achiral and chiral enstatite dimer structures using the CAM-B3LYP/cc-pVQZ and ωB97X-D3/cc-pVQZ levels of theory. From top to bottom, the left column shows Structures E, F, G, and H. Similarly, the right-hand column shows Structures I, J6, K, and L1. The spectra were created by using 60 transition states with a Gaussian line shape and a peak width of 24 nm. All of the structures show their major peak at about 150 nm except for J6, which shows major peaks at about 175 and 225 nm. The wavelengths with the strongest absorption correspond to energies that are high enough to break bonds.

UV–vis-NIR reflectance spectra for bulk enstatite indicate strong UV absorption below ∼400 nm caused by intense oxygen-to-metal charge transfer, which are centered at 200 and 300 nm;^112,113^ spectra of enstatite nanosilicates have not been studied previously. Figures 8 and 9 show the TD-DFT predicted UV–vis spectra of the monomer and dimer structures for two different functionals. No corrective energy shift was used for the spectra, as experimental data is not available for calibration; therefore, TD-DFT calculations can only qualitatively predict absorption features. Spectral results utilizing 60 transition states are shown (Figures 8 and 9), with a Gaussian line shape and a peak width of 24 nm.

There is a good qualitative agreement between the monomeric structure spectra calculated by utilizing the two different functionals. The protonated monomer UV–vis spectra are similar, with the CAM-B3LYP functional predicting a shoulder at ∼175 nm on the main ∼140 nm absorption feature. Quantitative disagreement largely came in the bare monomer, where the CAM-B3LYP functional predicted the second local maximum to occur at a slightly higher wavelength (∼270 nm) compared to (red-shifted) ωB97X-D3 (∼245 nm).

#### 3.3.2 Comparison of Dimer Conformers

The differences between each conformer type (e.g., the difference between an achiral dimer with a neutral hydrogen versus a chiral dimer with a neutral hydrogen) do not appear as large as when different hydrogenation types (e.g., H vs H^+^) are compared. The notable exception is the CAM-B3LYP/cc-pVQZ calculation for the proton. The calculation for an achiral dimer with a proton was drastically different from both ωB97X-D3 and the other conformer, with the results being significantly red-shifted. Furthermore, another peak at about 290 nm appears for the CAM-B3LYP functional for Structure G that is not present with the ωB97X-D3 functional nor the two analogous calculations for the chiral dimer (Structure K).

There is very little agreement, though, between the chiral dimer structure and its achiral version, showing that the linear portion of the ring created by one of the O–Mg–O fragments significantly affects the electronic structure of the molecule. Despite this, only the CAM-B3LYP/cc-pVQZ calculation is the outlier of all four calculations in terms of the overall shape of the spectra (Figure 9). The effect of the structure is more keenly seen in the 2D chiral dimer with a neutral H (Structure J5, which does not have 3D chirality), as the structure is most different from the others presented and produces a UV–vis curve distinct from the rest with two major peaks being nearly equal in strength.

While there is some agreement on the overall shape in the UV–vis spectra for the lowest energy enstatite dimers (Figure 9), the quantitative differences are considerable. There is no clear trend with CAM-B3LYP when it comes to the energies of the excited states between the two conformers: the highest excited state is higher for the achiral version, the second highest excited state energy is higher for the chiral version, and then higher again for the achiral version for the third highest excited state. The oscillator strengths also show no trend, and the transitions involve different orbitals. On the other hand, ωB97X-D3 predicts a universal red-shift for the chiral conformer energies compared to the achiral. The oscillator strength is stronger for the chiral version only with the second highest excited state. There is a disagreement again with the character of the transitions. Notably, the achiral conformer’s transitions originate from low-lying molecular orbitals.

It is difficult to pinpoint the reason for the disagreement when comparing the two functionals and then again when comparing the two dimer conformers. The latter’s analysis requires caution since the two functionals cannot even agree on the nature of the excited states. The prediction of different characters for excited states by two different functionals, such as CAM-B3LYP and ωB97X-D3, can arise due to several factors.

(1) Each functional is only an approximation of the true molecular electronic structure. While CAM-B3LYP and ωB97X-D3 are both density functional approximations used in TD-DFT calculations, each functional is based on different approximations and parametrizations, leading to variations in their accuracy and performance. CAM-B3LYP combines the Coulomb-attenuating method (CAM) with the B3LYP functional,^83^ while ωB97X-D3 incorporates long-range correction terms (D3) into the ωB97 functional.^85^ These modifications can affect how each functional treats excited states.
(2) The treatment of the exchange–correlation differs between the two functionals. CAM-B3LYP and ωB97X-D3 use different hybrid functional approaches, which combine a portion of exact (Hartree–Fock) exchange with density functionals.^83,85^ The specific mixing of exchange and correlation terms can influence the description of excited states.
(3) Sensitivity to the molecular environment can influence the electronic structure and excited states of molecules.^62^ CAM-B3LYP and ωB97X-D3 may respond differently to the surrounding molecular environment, leading to variations in the predicted characters of excited states.
(4) Some chemical systems respond better with one treatment versus another.^62^ This behavior can stem from the inherent strengths and weaknesses of the functional formulations, but without laboratory data to benchmark and test the predictability of the functionals, it is difficult to say which functional is more trustworthy. Future work could utilize experimental data or higher-level theoretical methods to identify the strengths and limitations of each of the functionals used here.

#### 3.3.3 Astronomical Implications of the Excited State Calculations

The identity of DIBs remains an unsolved riddle.^90^ While most of our results do not suggest that these hydrogenated nanosilicates could be the carriers of the majority of features, there is one DIB in the ultraviolet at 141.6 nm^114^ that might correspond to the studied nanosilicates (whether hydrogenated or bare). However, the feature might not have been above the noise level.^114,115^ This region of the electromagnetic spectrum continues to require more sensitive techniques to achieve firmer detections.

The absorption of light of the studied nanosilicates is predicted to occur at energies large enough (>5.05 eV) to break Si–O bonds.^116^ This implies that these structures could be important intermediates in the formation of larger structures through photochemistry. The reaction barriers and pathways remain to be elucidated. Structures J1–J4, J6, and J7 (Figure 3), along with structures L2 and L3, the three-dimensional chiral structures studied here, may preferentially absorb one-handedness of light, leading to enantioselective effects.

#### 3.3.4 Circular Dichroism Predictions and Implications for Chiral Bias

**Figure 10:**
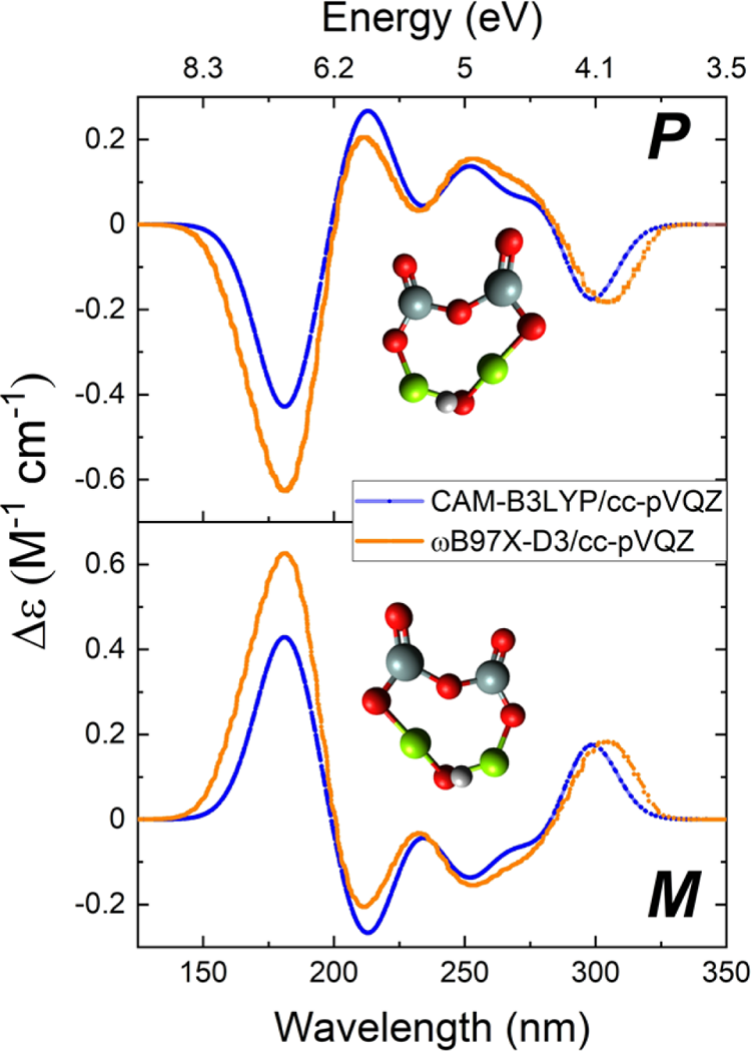
Calculated circular dichroism (CD) spectra of structure J1, the three-dimensional chiral structure with neutral hydrogen with the largest g-factor. The CD spectra for each enantiomer are shown. Both levels of theory predict qualitatively similar curves. These TD-DFT calculations suggest that a change in the absorption of the handedness of light changes after 175 nm but switches back after about 260 nm. The effect is relatively small but shows that, in principle, it is possible to break the symmetry early on in the formation of enstatite through asymmetrical absorption of circularly polarized light on enstatite nanosilicates. The ωB97X-D3 functional was shifted by −2.8 nm to match the local maxima and minima of the two functionals.

Figure 10 and Table 10 give information about the electronic circular dichroism (ECD) of the chiral HI-enstatite dimer (Structure J1, which showed the greatest enantioselectivity). Figures S5 and S6 show the UV–vis and ECD spectra for the axially chiral structures and those with a stereogenic atom, respectively. Table 10 also shows the wavelengths where at least one of the computational methods predicted a *g*-value of at least 0.001. Circularly polarized light interacting with the *P*- and *M*-enantiomers absorbs the UV-CPL equally in magnitude but in the opposite direction. Since no functional group within the chiral dimer rotates, Boltzmann averaging was not used for the ECD calculations. The effects predicted by the CAM-B3LYP/cc-pVQZ and ωB97X-D3/cc-pVQZ levels of theory are similar, but the appearance of certain features with the CAM-B3LYP functional lags behind ωB97X-D3. A small energy shift (∼2.8 nm) for the ωB97X-D3 was conducted to better compare simulated spectral data in Figure 9. The variation between results is likely due to different parametrizations and approximations in the calculation. CAM-B3LYP was specifically developed for the calculation of electronic excitation energies in molecules that B3LYP struggles to accurately describe,^83^ while ωB97X-D3 was designed to perform well in a broad range of chemical systems.^85^ Both functionals (or their families) have been used in recent work to successfully compute properties of silicates^117^ and silica clusters.^118,119^

**Table 10:** Calculated *g*-Factors of the Chiral Molecules for Local Maxima/Minima

		λ (nm)		|Δε| (M^–1^ cm^–1^)		ε (M^–1^ cm^–1^)		|*g*| factor	
structure	local max/min	CAM-B3LYP/cc-pVQZ	ωB97x-D3/cc-pVQZ	CAM-B3LYP/cc-pVQZ	ωB97x-D3/cc-pVQZ	CAM-B3LYP/cc-pVQZ	ωB97x-D3/cc-pVQZ	CAM-B3LYP/cc-pVQZ	ωB97x-D3/cc-pVQZ
J1	λ_1_	181	171	0.428	0.626	286	476	0.001	0.001
	λ_2_	--	207	--	0.159	--	121	--	0.001
	λ_4_	265	253	0.0797	0.105	22	16	0.004	0.007
	λ_5_	292	282	0.131	0.182	136	164	0.001	0.001
J2	λ_2_	200	194	0.104	0.284	241	262	0.0004	0.001
	λ_4_	262	247	0.0140	0.143	20	64	0.001	0.001
	λ_6_	336	296	0.00198	0.00926	1	21	0.003	0.0004
J3	λ_4_	262	259	0.0484	0.0412	74	71	0.001	0.001
J4	λ_1_	192	181	0.219	0.358	242	287	0.001	0.001
	λ_3_	229	217	0.275	0.169	267	280	0.001	0.001
J6	λ_2_	200	193	0.0911	0.126	116	163	0.001	0.001
	λ_4_	260	239	0.130	0.197	26	58	0.005	0.003
J7	λ_2_	211	199	0.198	0.184	159	158	0.001	0.001
	λ_4_	269	246	0.0416	0.165	18	52	0.002	0.003
	λ_6_	--	309	--	0.0101	--	3	--	0.003
	λ_7_	--	328	--	0.0236	--	7	--	0.004
L2	λ_2_	206	197	0.213	0.283	137	158	0.002	0.002
	λ_4_	261	242	0.0138	0.0160	7	30	0.002	0.001
L3	λ_1_	155	150	0.105	1.28	642	792	0.0002	0.002
	λ_2_	205	196	0.203	0.244	142	157	0.001	0.002
	λ_4_	260	241	0.0213	0.0221	8	28	0.003	0.001

The *g*-factor, also called the anisotropy or dissymmetry factor, represents the ratio between the circular dichroism (Δε) to the extinction coefficient (ε). These *g*-factors provide a way of standardizing the results from the ECD calculations to the overall absorbance of a molecule. Table 10 shows the magnitudes of the calculated *g*-factors (*g* = |Δε|/ε) for the local maxima or minima for both levels of theory in the chiral structures. The largest is 0.004/0.007 (depending on the theory used), which is relatively small considering that *g*-factors can have an absolute value of up to 2. This does not represent a large divergence in results, and the consistency between methods suggests that the asymmetric absorption is real.

For comparison, g-values for neutral amino acids like (chiral) alanine are ∼0.007.^120^ However, *g*-factors may be much greater for charged chiral amino acids, ranging 0.028–0.040.^121,122^ While the calculated maximum *g*-factors for the chiral dimer with HI (Structure J1) are small, the nanosilicate molecule can be found to be much closer to the protostar in a protoplanetary disk. Since the intensity of light decays by the inverse square of distance, the amount of light received by the nanosilicates will be much larger than the light received by the amino acids. This could compensate for the relatively low g-factors of the studied nanosilicates compared to amino acids.

Overall, the asymmetric absorption of UV-CPL, as measured by the g-factor, is small. However, since we describe a nanosilicate dimer of only ∼10 Å, this is not entirely unexpected, as there is a strong size dependence between nanoparticles and the dissymmetry factor, in addition to other parameters like shape.^123^ For example, gold nanoparticles of ∼42 nm had *g*-factors on the order of 0.004,^124^ on the same order as Structures J1, J2, J6, and J7. Larger versions of the gold nanoparticles (up to 180 nm) were able to obtain higher *g*-factors (∼0.2) that indicate a stronger preferential interaction with CPL.^123^ As size and shape are important determinants of the strength of asymmetrical UV absorption, larger chiral nanosilicates may show similar *g*-factor size enhancement.

Whether seeding a chiral preference in enstatite in the ISM can lead to an overall enantiomeric bias in bulk clinoenstatite remains unconfirmed. One possibility is a down–up mechanism, where the absorption of HI, abundant in interstellar space,^125^ leads to the formation of enstatite chiral HI-dimers, a structure with axial chirality. Adding additional chiral enstatite subunits could lead to an inherently helical structure. Thus, preferential destruction by UV-CPL of one magnesium nanosilicate enantiomer has the potential to produce enhancement in one putative helical structure over another. Other helical structures like triple helical nanowires, helical perovskite nanocrystals, and helical silicon nanowire multilayers exhibit strong *g*-values of up to 0.75,^126^ 0.02,^127^ and 1.17,^128^ respectively. It is interesting to note that there is evidence of such helical and axial chirality in enstatite structures in meteorites and dust particles in the form of nanoribbons and screw dislocations.^129−131^ CdTe ribbons have previously been shown to result in an enantiomeric excess above 30% when interacting with CPL,^58^ and it is plausible that enstatite nanoribbons could also have certain enantiomorphs preferred for formation or destruction by CPL.

In the ISM or other interstellar environment, helical structures may preferentially enhance one chiral enantiomorph (*P*- or *M*-) for interaction with chiral organic precursors and molecules. Chiral indices calculated for diopside (MgCaSi_2_O_6_), a related pyroxene mineral, are high, suggesting that the chiral face would have a strong enantioselective effect on organics.^132^

## 4 Conclusions

Nanosilicates represent an understudied class of molecule in astrochemistry and cosmochemistry, particularly enstatite, a magnesium silicate (pyroxene) whose formation in protoplanetary disks and the ISM is not fully understood. An examination of both the bare and hydrogenated enstatite nanosilicates found the majority were achiral, with the exception of one 2D chiral dimer that exhibited three-dimensional chirality with the addition of a neutral H. The hydrogenated forms of the nanoenstatite structures appear more stable than the unhydrogenated forms, a result similar to hydroxylated nanosilicates.^28^ The predicted IR modes suggest that both bare and hydrogenated forms of magnesium nanosilicate molecules should produce strong features at molecule-specific wavelengths within the observable range (∼1 to 28 μm) of the JWST.

Identification of the many diffuse interstellar bands (DIBs) and unidentified infrared emission bands (UIBs)^90^ represents two outstanding puzzles in astrochemistry. While DIB features are generally attributed to PAHs, only C_60_^+^ has been identified specifically,^99^ leaving hundreds of DIBs that remain unidentified.^90,100^ Furthermore, tying a particular set of PAHs to UIBs has proven to be difficult. Nanosilicates, with vibrational and electronic absorption features covering a vast spectral range, are likely to be present in the diffuse ISM and have the potential to contribute to both DIB and UIB signals. In particular, we find overlap with UIB bands in optical absorption features for most of our calculated magnesium nanosilicate monomers and dimers, particularly at the 7.7 μm (1298.7 cm^–1^) feature, usually attributed to C–C stretching from PAHs; the 6.3 μm (1587.3 cm^–1^), also attributed to C–C stretching; and the 3.3 μm feature (3030.3 cm^–1^), normally attributed to aromatic C–H stretching. Other hydrogenated nanosilicates may show similar patterns of behavior and represent a broad class of molecules that require deeper investigation.

To the best of our knowledge, no TD-DFT calculations on astrophysically relevant silicates have been previously published. We found that CAM-B3LYP/cc-pVQZ and ωB97X-D3/cc-pVQZ levels of theory predict UV absorption at energies of about 8 eV or larger for the chiral hydrogenated dimer, which can be conducive to forming even larger enstatite nanosilicates or recycling the species to form new products through photochemical processing. For the bare and hydrogenated chiral enstatite dimers, the TD-DFT calculations predict a small (*g*-factors up to 0.007) asymmetry in the absorption of UV-CPL, where larger chiral nanosilicates are likely to be affected more strongly. However, we emphasize that the specific base dimer silicates studied in this investigation should only be taken as a “proof-positive” test case for enantioselective astrochemistry in the ISM, where calculations demonstrate the potential stability of chiral nanosilicate structures and their ability to be selectively acted upon with circularly polarized light. As a high-energy isomer is utilized to create the chiral compounds, these are unlikely to be abundant in the ISM.

The ECD implications for terrestrial homochirality are clear. If chiral minerals act on chiral organic molecules within protoplanetary disks, then this process could be responsible for the enantiomeric excess of left-handed amino acids and right-handed sugars seen in carbonaceous meteorites. Bulk minerals like quartz (SiO_2_) and calcite (CaCO_3_), along with high-index metals, have been studied for their enantioselectivity with positive results.^47−54^ However, chiral surfaces of terrestrial minerals like quartz have been found to be racemic on Earth^133^ and do not make up a large contribution to meteorites.^44^ Quantitative measurement of asymmetries in chiral mineral surfaces present in meteoritic, asteroidal, or cometary materials is needed to ascertain the efficacy of this enantioselective mechanism.

## Supporting Information Available

Benchmarking work, energy calculations, structure files, frequency data, and all computed electronic transition states can be found in the supplementary work (PDF)
