# Supplementary material for: Search for Chirality in Hydrogenated Magnesium Nanosilicates: A DFT and TD-DFT Investigation
Source: J Phys Chem A. 2024 Apr 30;128(18):3475–94. doi: 10.1021/acs.jpca.3c06521 (PMC11089509; doi:10.1021/acs.jpca.3c06521)
Supplement: Supplementary file 1 — jp3c06521_si_001.pdf [file jp3c06521_si_001.pdf]

Supporting Information

A Search for Chirality in Hydrogenated Magnesium Nanosilicates: A DFT and TD-DFT Investigation

Kamil B. Stelmach[1,2*], Catherine A. Dukes[2], and Robin T. Garrod[1,3]

[1] Department of Chemistry, University of Virginia, Charlottesville, VA 22904

[2]Laboratory for Astrophysics and Surface Physics, Department of Materials Science and Engineering, University of Virginia, Charlottesville, VA 22904

[3] Department of Astronomy, University of Virginia, Charlottesville, VA 22904

*Corresponding Author: Kamil Stelmach (kbs7dqw@virginia.edu)

S.1 Benchmarking

The choice of functional was an important step before moving forward with the calculations. The functionals were benchmarked for the bare achiral monomer and dimer against the CCSD(T)+F12 calculations performed in Valencia et al. (1) with HSE06/aug-cc-pVQZ providing the best results. The optimization (bond lengths and bond angles) and frequencies for the monomer were used for comparison (Tables S.1 and S.2) along with the energy calculations of the dimers (Table S.3). The B3LYP/aug-cc-pVQZ level of theory performed best in the optimization calculations based on various statistical tests but was only marginally better than HSE06/aug-cc-pVQZ (0.00394 Å average deviation from bond length for B3LYP compared to 0.05540 Å for HSE06 and 0.13466° average deviation in the bond angle for B3LYP whereas HSE06 had 0.16813°). However, the latter level of theory better predicted the harmonic frequencies of the enstatite monomer (2.76 cm^{-1} average deviation for HSE06 whereas B3LYP had 10.24 cm^{-1}) so this combination of functional and basis set was used for the DFT calculations. The energy calculations (Table S.3) were also closer in agreement to CCSD(T)+F12 compared to B3LYP (0.1593 a.u. average percent deviation for the former and 1.635 a.u. for the latter).

Table S.1 shows the optimization comparison of three hybrid functionals, namely: B3LYP, B3PW91, and HSE06. Basis functions tested included cc-pVQZ, aug-cc-pVQZ, and DGTZV. DGTZV gave the poorest agreement to the CCSD(T)+F12 calculations whereas aug-cc-pVQZ gave the closest approximations albeit at a slightly higher computational cost.

While B3LYP/aug-cc-pVQZ did marginally better in duplicating the bond angles and distances for the enstatite monomer, Table S.2 shows that HSE06/aug-cc-pVQZ matched the frequency calculations from CCSD(T)+F12 more closely. Both B3LYP and HSE06 are commonly used exchange-correlation functionals in DFT. While B3LYP is a popular functional, it has been known to overestimate bond lengths and underestimate bond angles.

HSE06, on the other hand, is a range-separated hybrid functional that includes both short-range and long-range interactions. It has been shown to improve the description of van der Waals interactions and has been demonstrated to perform better than B3LYP in optimizing geometries (2) and calculating energies (3, 4) of large molecules and inorganic materials.

Similarly, Tables S.3 and S.4 show that calculations utilizing HSE06/aug-cc-pVQZ best match the energy calculations using CCSD(T)+F12.a higher level of theory. In terms of single-point energy calculations, HSE06 is generally expected to be more accurate than B3LYP (3, 4). This is because HSE06 uses a hybrid functional that includes a higher percentage of Hartree-Fock exchange than B3LYP, which typically leads to better accuracy for energy calculations. Additionally, HSE06 includes a correction term to account for long-range electron interactions, which can be particularly important for accurate calculations of large molecules or systems with weak intermolecular interactions.

Table S1 Geometry Calculation Comparisons for the Enstatite Monomer

Theory	Functional	B3LYP		B3PW91		HSE06	
	Basis Set	cc-pVQZ	aug-cc-pVQZ	cc-pVQZ	aug-cc-pVQZ	cc-pVQZ	aug-cc-pVQZ
Bond Length	Average Deviation	0.00404	0.00394	0.00570	0.00553	0.00570	0.05540
	Standard Deviation	0.00234	0.00215	0.17209	0.15819	0.00484	0.00573
Bond Angles	Average Deviation	0.14427	0.13466	0.25020	0.22322	0.25027	0.16813
	Standard Deviation	0.88836	0.05762	0.19528	0.18466	0.19519	0.16128

Table S2 Frequency Calculation Comparisons with CCSD(T)-F12/cc-pVTZ-F12 (Valencia et al., 2020) Using Percent Average Deviation, Standard Deviation, Coefficient of Variation (CV), and the Mean Absolute Error (MAE) for the Enstatite Monomer

Theory	Functional	B3LYP			B3PW91			HSE06		
	Basis Set	cc-pVQZ	aug-cc-pVQZ	DGTZVP	cc-pVQZ	aug-cc-pVQZ	DGTZVP	cc-pVQZ	aug-cc-pVQZ	DGTZVP
Statistical Test	Average % Deviation	9.86	10.24	21.9	4.52	5.21	16.9	3.47	2.76	13.6
	Standard Deviation	7.27	6.33	11.7	3.65	3.41	9.37	2.63	1.48	7.66
	CV	0.738	0.619	0.535	0.806	0.655	0.554	0.760	0.536	0.562
	MAE	1.10	1.14	2.44	0.502	0.579	1.88	0.385	0.307	1.514

Table S3 Energy Calculation Comparisons with CCSD(T)-F12/cc-pVTZ-F12 (Valencia et al., 2020) Using Percent Average Deviation, Standard Deviation, Coefficient of Variation (CV), and the Mean Absolute Error (MAE) for the Enstatite Dimers

Theory	Functional	B3LYP		B3PW91		HSE06	
	Basis Set	cc-pVQZ	aug-cc-pVQZ	cc-pVQZ	aug-cc-pVQZ	cc-pVQZ	aug-cc-pVQZ
Statistical Test	Average % Deviation	1.559	1.635	0.4235	0.4871	1.470	0.1593
	Standard Deviation	3.872	3.963	0.9553	1.033	3.678	0.2722
	CV	2.483	2.424	2.256	2.121	2.502	1.709
	MAE	0.5197	0.5449	0.141	0.1624	0.4901	0.0531

Table S4 Unhydrogenated Dimer Energy Calculations

Dimer Structure	HSE06/aug-cc-pVQZ (a.u.)	Relative Energies (kcal mol^{-1})
Valencia et al. (2020) B	-1430.425006	3.581
Valencia et al. (2020) D	-1430.374492	35.279
Valencia et al. (2020) E	-1430.357942	45.663
Valencia et al. (2020) F	-1430.430713	0.000
Valencia et al. (2020) G (Achiral Conformer – Structure I in this Paper)	-1430.327309	64.886
Chiral Conformer – Structure E in this Paper	-1430.327294	64.895

S.2 Placement of Adsorbates in Calculations

The location of the adsorbates is shown in Figure S.1. Equivalent positions from symmetry are marked in red and were not calculated (e.g. position 1 is equivalent to position 3). Positions in parentheses represent positions in a plane a couple angstroms above the plane of each molecule.

S.3 Molecular Stability

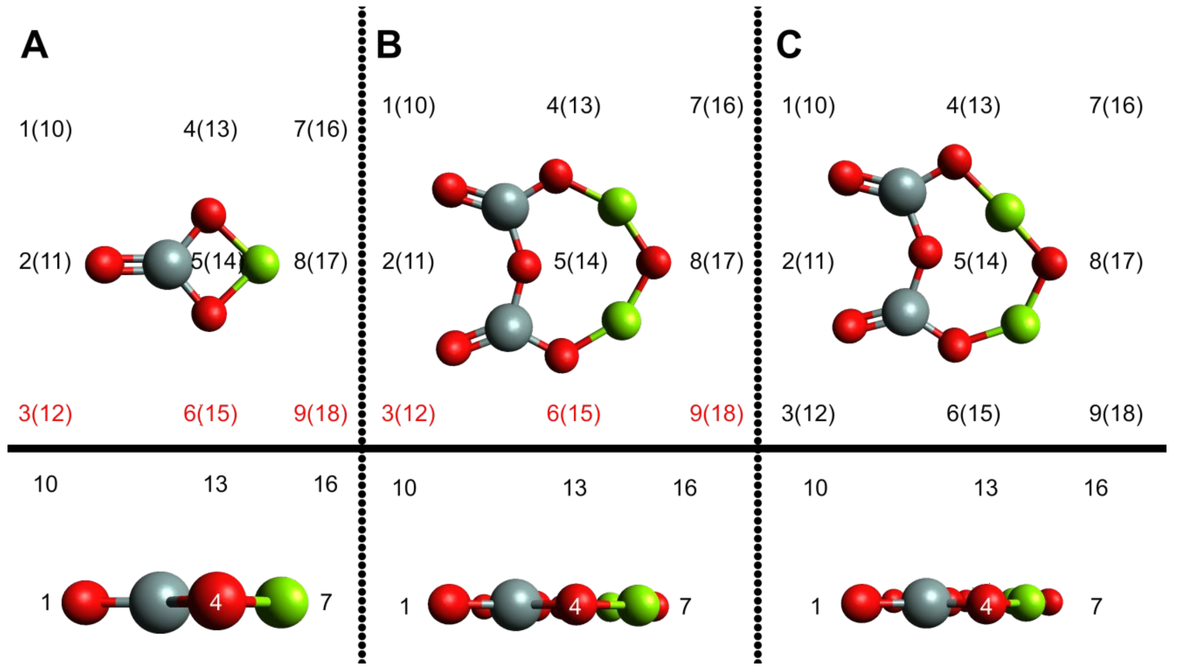

Figure S1
The placement of adsorbate on the (A) monomer, (B) achiral dimer, and (C) chiral dimer. The top view shows the ring of each species and the bottom view shows the orthogonal view, revealing the planar structures. Positions in red were not calculated due to being equivalent to other calculated positions. Numbers in parentheses represent placement above the plane of the molecule. Oxygen atoms are red, silicon atoms are grey, magnesium atoms are green, and hydrogen atoms are white.

HOMO-LUMO gaps can be used to predict molecular stability with larger gaps usually being associated with a more stable compound. Figures S.2-S.4 show the HOMO and LUMO gaps of the monomer and chiral dimers, respectively. The DFT calculations with HSE06/aug-cc-pVQZ on enstatite dimers reveal that all the HOMO-LUMO gaps are < 4.5 eV, similar to Reber et al. (5) who reported values 0.75 eV to 4.75 eV for nanosilicates containing SiO. This relatively small energy difference between the HOMO and the LUMO suggests that the system is more prone to electron transfer reactions, useful for photocatalytic reactions as they possess suitable band gaps for absorption of visible light.

The frontier orbitals' energies provide information concerning molecular kinetic stability of the molecules; the larger the HOMO-LUMO gap, generally the more stable and less reactive a molecule appears. For the nanosilicate enstatite studied in this work, there was no consistent trend. In the case of the monomers (Figure 1 from the main text), the most stable molecule has H_2 adsorbed. The monomer with the neutral H and the monomer with the H^+ have similar predicted stabilities from the SOMO-LUMO and HOMO-LUMO gaps, respectively. However, the HOMO and LUMO orbitals for the protonated monomeric enstatite (Structure C1) are lower in energy compared to that of hydrogenated

enstatite (Structure B1), and the LUMO orbitals are lower in energy than each of the HOMO or SUMO orbitals for the rest of the structures. The most reactive molecule is predicted to be the unhydrogenated dimer.

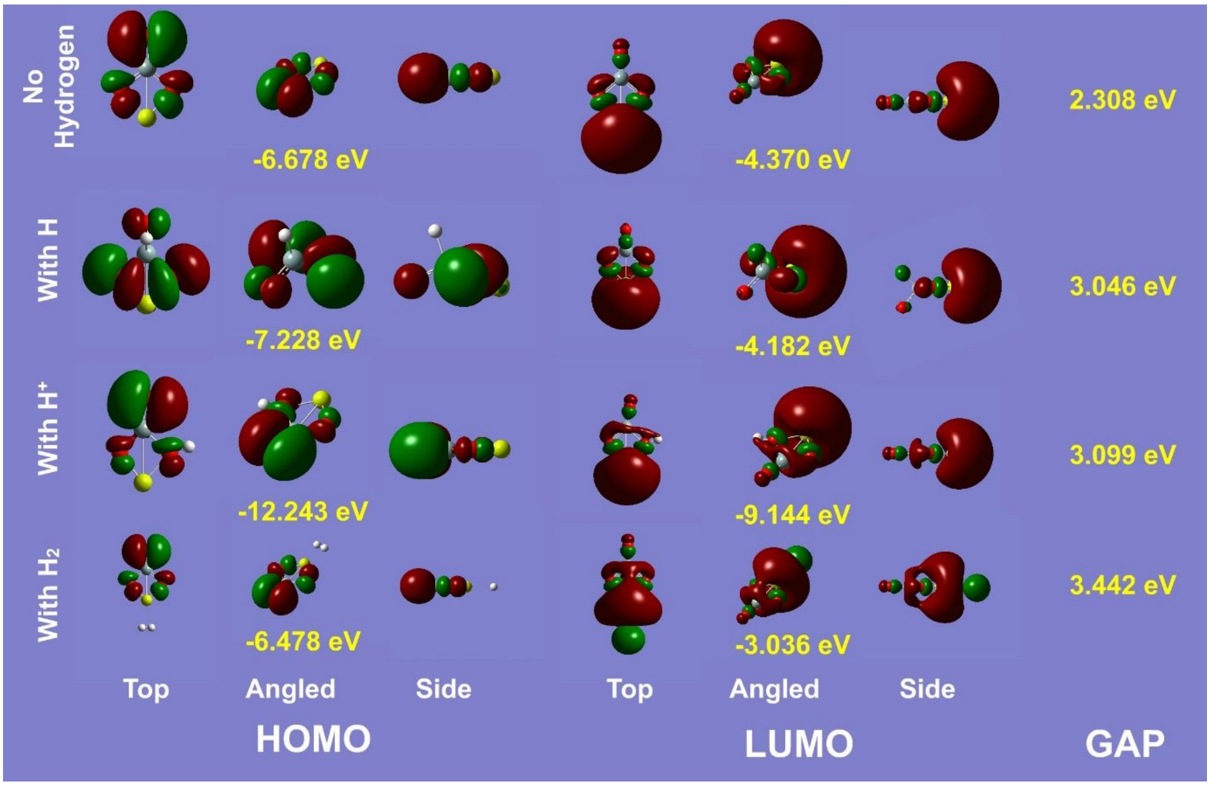

Figure S2 The predicted HOMO, LUMO, and their respective energy gap from HSE06/aug-cc-pVQZ for all the different monomer structures (Structures A, B, C, and D), which were optimized using the same level of theory. Green and red orbitals represent the positive and negative regions of the MO, respectively. Each molecular orbital type is show from a bird's eye view, from the side such that the structures are either planar or mostly planar, and from angle such that the silicon atom is closer to the viewer than the magnesium atom. The frontier orbitals for the structures with neutral hydrogen actually represent the SOMO-LUMO gap.

For the achiral dimers, the unhydrogenated and complex with H_2 adsorbed were most stable (Figure S.3). Achiral enstatite dimers with a neutral H and H^+ (Structures F and G, respectively) show similar HOMO-LUMO gaps. However, as with the monomer, the energies of the HOMO and LUMO orbitals for the structure with the proton are much lower (~3 eV) than that of the neutral H or any other structure. The stabilities of the bare chiral dimer and that with H_2 are reversed, with the protonated enstatite dimer (Structure L1) being the least stable. Complexes with neutral H and a proton (Structure J1 and K, respectively) show similar stabilities, although the LUMO of the chiral enstatite with H is lower in energy than any of the HOMO/SUMO values for the rest of the structures.

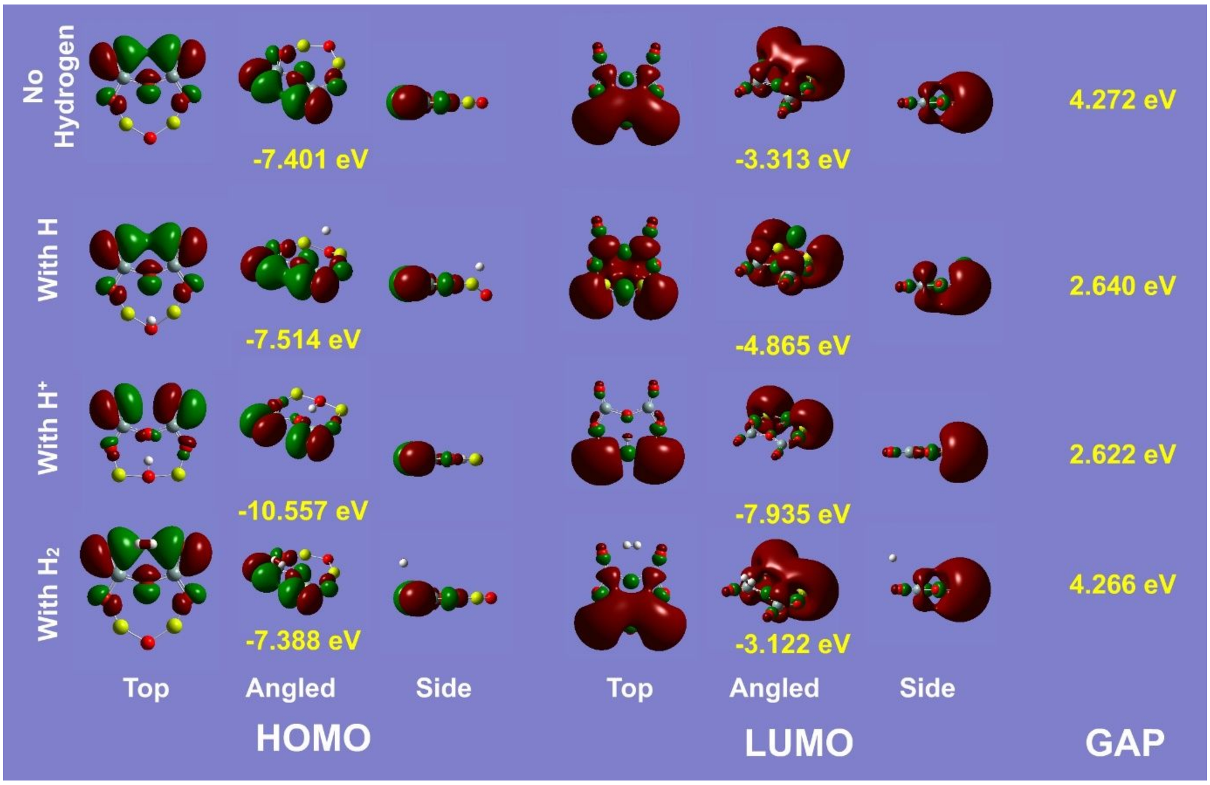

Figure S3 The predicted HOMO, LUMO, and their respective energy gap from HSE06/aug-cc-pVQZ for all the different achiral structures (Structures E, F, G, and H), which were optimized using the same level of theory. Green and red orbitals represent the positive and negative regions of the MO, respectively. Each molecular orbital type is show from a bird's eye view, from the side such that the structures are either planar or mostly planar, and from angle such that the silicon atom is closer to the viewer than the magnesium atom. The frontier orbitals for the structures with neutral hydrogen actually represent the SOMO-LUMO gap.

For protonated achiral complexes, the electronic structure for this grouping is lower in energy than the rest. The lower energy HOMO level indicates it is more likely to donate electrons, whereas lower energy LUMO level indicates that the molecule is more likely to accept electrons, implying enhanced reactivity compared with other molecules studied here. Cationic species like this are common in astrochemistry for building up larger molecules, especially molecules that contain H^+. The prototypical example is H_3^+, which is responsible for forming the precursors to species observed in dense molecular clouds like CH^+ and CH_2^+ (6-8). Another example of cationic species utilizing hydrogen to build up the size of molecules includes carbon chains (9).

While reaction dynamics were not explored in this study, it would be interesting to investigate the complex formation between the various enstatite nanosilicates and the different forms of hydrogen to determine the size of the reaction barriers, if any, for forming such complexes. Second, the reaction

dynamics of dimer synthesis has not yet been elucidated. Finally, the reactions between dimers to form even larger complexes (bulk) silicates should be determined.

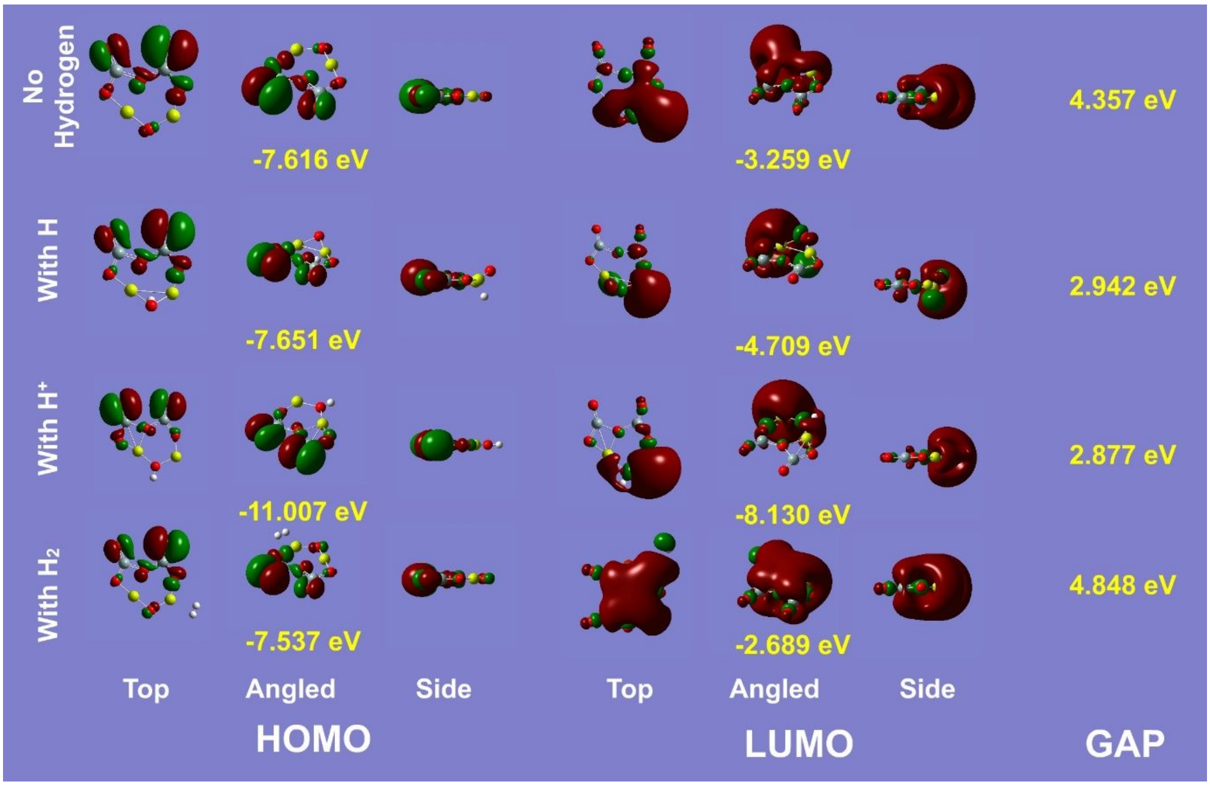

Figure S4 The predicted HOMO, LUMO, and their respective energy gap from HSE06/aug-cc-pVQZ for all the different chiral structures (Structures I, J, K, and L), which were optimized using the same level of theory. Green and red orbitals represent the positive and negative regions of the MO, respectively. Each molecular orbital type is show from a bird's eye view, from the side such that the structures are either planar or mostly planar, and from angle such that the silicon atom is closer to the viewer than the magnesium atom. The frontier orbitals for the structures with neutral hydrogen actually represent the SOMO-LUMO gap.

Table S5 Energy Calculations of All Molecules

Structure	Description	Energy (a.u.)
n/a	H	-0.501444526780
n/a	H^+	0.000
n/a	H_2	-1.16915299734
A	$MgSiO_3$	-715.094400
B1	$MgSiO_3 + H$	-715.641691
B2		-715.727365
B3		-715.638672
B4		-715.712427
C1	$MgSiO_3 + H^+$	-715.442899
C2		-715.482833
D1	$MgSiO_3 + H_2$	-716.274547
E	Achiral $Mg_2Si_2O_6$	-1430.327309
F	*Achiral* $Mg_2Si_2O_6 + H$	-1430.853349
G1	Achiral $Mg_2Si_2O_6 + H^+$	-1430.650960
H	*Achiral* $Mg_2Si_2O_6 + H_2$	-1431.497589
I	Chiral $Mg_2Si_2O_6$	-1430.327294
J	Chiral $Mg_2Si_2O_6 + H$	-1430.854478
K	Chiral $Mg_2Si_2O_6 + H^+$	-1430.685802
L	Chiral $Mg_2Si_2O_6 + H_2$	-1431.505497

Table S6 Atomic Positions (MOL2) of the Monomer – Structure A

1 Si1	0.0000	0.0000	0.6729 Si
2 O2	0.0000	0.0000	2.1912 O
3 O3	0.0000	1.2563	-0.3768 O
4 O4	0.0000	-1.2563	-0.3768 O
5 Mg5	0.0000	0.0000	-1.7435 Mg

Table S7 Atomic Positions (MOL2) of the Monomer + H – Structure B1

1Si1	-0.6442	0.0000	0.3358 Si
2 O2	-2.0175	0.0000	-0.6028 O
3 O3	0.3857	-1.2643	0.0900 O
4 O4	0.3857	1.2643	0.0900 O
5 Mg5	1.6936	0.0000	-0.2480 Mg
6 H6	-1.3366	0.0001	1.6569 H

Table S8 Atomic Positions (MOL2) of the Monomer + H – Structure B2

1 Si1	-0.5716	-0.0254	0.0000 Si
2 O2	-2.1778	-0.095	0.0000 O
3 O3	0.342	-1.2866	0.0000 O
4 O4	0.2783	1.2844	0.0000 O
5 Mg5	1.9261	0.0329	0.0000 Mg
6 H6	-2.6513	0.7391	0.0000 H

Table S9 Atomic Positions (MOL2) of the Monomer + H – Structure B3

1 Si1	-0.6944	0.0000	0.0000 Si
2 O2	-2.3133	0.0000	0.0000 O
3 O3	0.1832	1.2836	0.0000 O
4 O4	0.1832	-1.2836	0.0000 O
5 Mg5	1.8159	0.0000	0.0000 Mg
6 H6	3.5055	0.0000	0.0000 H

Table S10 Atomic Positions (MOL2) of the Monomer + H – Structure B4

1 Si1	0.7483	-0.1458	0.0000 Si
2 O2	2.2593	-0.0732	0.0001 O
3 O3	-0.1972	1.2558	0.0000 O
4 O4	-0.4364	-1.1762	-0.0001 O
5 Mg5	-1.9753	-0.0107	-0.0001 Mg
6 H6	0.2227	2.1179	0.0001 H

Table S11 Atomic Positions (MOL2) of the Monomer + H^+ – Structure C1

1 Si1	0.7481	-0.1641	0.0000 Si
2 O2	2.2366	0.0242	0.0000 O
3 O3	-0.3299	1.2139	0.0000 O
4 O4	-0.4471	-1.2068	0.0000 O
5 Mg5	-1.8483	-0.0051	0.0000 Mg
6 H6	0.0288	2.1078	0.0001 H

Table S12 Atomic Positions (MOL2) of the Monomer + H^+ – Structure C2

1 Si1	0.0000	0.5767	0.0000 Si
2 O2	-0.0598	2.1509	0.0000 O
3 O3	-1.2619	-0.3696	0.0000 O
4 O4	1.2556	-0.3842	0.0000 O
5 Mg5	-0.0154	-1.831	0.0000 Mg
6 H6	0.7134	2.7214	0.0001 H

Table S13 Atomic Positions (MOL2) of the Monomer + H_2 – Structure D1

1 Si1	-0.8230	0.0000	-0.0001 Si
2 O2	-2.3418	0.0000	-0.0003 O
3 O3	0.2242	1.2560	0.0002 O
4 O4	0.2242	-1.2560	-0.0001 O
5 Mg5	1.5971	0.0000	0.0002 Mg
6 H6	3.7520	-0.3780	0.0005 H
1 H7	3.7520	0.3780	0.0006 H

Table S14 Atomic Positions (MOL2) of the Monomer + H_2 – Structure D2

1 Si1	-0.8230	0.0000	-0.0001 Si
2 O2	-2.3418	0.0000	-0.0003 O
3 O3	0.2242	1.2560	0.0002 O
4 O4	0.2242	-1.2560	-0.0001 O
5 Mg5	1.5971	0.0000	0.0002 Mg
6 H6	3.7520	-0.3780	0.0005 H
1 H7	3.7520	0.3780	0.0006 H

Table S15 Atomic Positions (MOL2) of the Achiral Dimer – Structure E

1 Si1	-1.0611	1.5886	0.0000 Si
2 O2	-2.4943	2.0677	0.0000 O
3 O3	-0.6078	0.0000	-0.0000 O
4 O4	0.3089	2.3872	0.0000 O
5 O5	0.3089	-2.3872	0.0000 O
6 O6	-2.4943	-2.0677	-0.0000 O
7 O7	2.8782	-0.0000	0.0000 O
8 Mg8	1.9380	1.5288	0.0000 Mg
9 Mg9	1.9380	-1.5288	0.0000 Mg
10 Si10	-1.0611	-1.5886	-0.0000 Si

Table S16 Atomic Positions (MOL2) of the Achiral Dimer + H – Structure F

1 Si1	-0.0068	1.0642	1.5805 Si
2 O2	-0.0685	2.5129	2.0030 O
3 O3	-0.0033	0.5483	0.0000 O
4 O4	0.0669	-0.2877	2.4035 O
5 O5	0.0669	-0.2877	-2.4035 O
6 O6	-0.0685	2.5129	-2.0030 O
7 O7	-0.5486	-2.8499	-0.0000 O
8 Mg8	0.1369	-1.8613	1.4447 Mg
9 Mg9	0.1369	-1.8613	-1.4447 Mg
10 Si10	-0.0068	1.0642	-1.5805 Si
11 H11	1.3445	-2.3166	-0.0000 H

Table S17 Atomic Positions (MOL2) of the Achiral Dimer + H^+ – Structure G

1 Si1	0.0000	-1.5860	-1.0414 Si
2 O2	0.0000	-2.0158	-2.4824 O
3 O3	0.0000	0.0000	-0.5202 O
4 O4	0.0000	-2.4056	0.3248 O
5 O5	0.0000	2.4056	0.3248 O
6 O6	0.0000	2.0158	-2.4824 O
7 O7	0.0000	0.0000	2.1721 O
8 Mg8	0.0000	-1.8948	2.0539 Mg
9 Mg9	0.0000	1.8948	2.0539 Mg
10 Si10	0.0000	1.5860	-1.0414 Si
11 H11	0.0000	0.0000	1.1692 H

Table S18 Atomic Positions (MOL2) of the Achiral Dimer + H_2 – Structure H

1 Si1	-0.0978	0.9990	1.5899 Si
2 O2	-0.1578	2.4273	2.0808 O
3 O3	-0.0970	0.5549	0.0000 O
4 O4	-0.0258	-0.3721	2.3831 O
5 O5	-0.0258	-0.3721	-2.3831 O
6 O6	-0.1578	2.4273	-2.0808 O
7 O7	0.0971	-2.9393	0.0000 O
8 Mg8	0.0512	-2.0010	1.5292 Mg
9 Mg9	0.0512	-2.0010	-1.5292 Mg
10 Si10	-0.0978	0.9990	-1.5899 Si
11 H11	2.2232	3.1223	0.3731 H
12 H12	2.2232	3.1223	-0.3731 H

Table S19 Atomic Positions (MOL2) of the Chiral Dimer – Structure I

1 Si1	0.3193	1.7207	-0.0002 Si
2 O2	-0.4248	0.2275	0.0000 O
3 O3	-0.4554	3.0171	-0.0002 O
4 O4	1.8727	1.4284	-0.0003 O
5 Mg5	2.7226	-0.2046	-0.0002 Mg
6 Si6	-1.9626	-0.4633	0.0002 Si
7 O7	-1.5550	-1.9912	0.0003 O
8 O8	-3.2533	0.3185	0.0002 O
9 O9	2.1251	-1.8897	0.0000 O
10 Mg10	0.3216	-2.0026	0.0002 Mg

Table S20 Atomic Positions (MOL2) of the Chiral Dimer + H – Structure J1

1 Si1	-0.2402	1.7383	-0.0320 Si
2 O2	0.4430	0.2113	-0.0056 O
3 O3	0.5872	2.9918	-0.1853 O
4 O4	-1.7949	1.5025	0.1119 O
5 Mg5	-2.6506	-0.1246	0.2223 Mg
6 Si6	1.9592	-0.5405	0.0234 Si
7 O7	1.4733	-2.0445	0.0371 O
8 O8	3.2765	0.1942	0.0479 O
9 O9	-2.1884	-1.7603	-0.5618 O
10 Mg10	-0.3998	-1.8831	0.0466 Mg
11 H11	-1.8344	-1.4353	1.3402 H

Table S21 Atomic Positions (MOL2) of the Chiral Dimer + H – Structure J2

1 Si1	0.1524	1.6908	0.4522 Si
2 O2	-0.3191	0.0279	0.4541 O
3 O3	-0.8983	2.4161	-0.5656 O
4 O4	1.6831	1.7057	-0.0071 O
5 Mg5	2.6135	0.1258	-0.2132 Mg
6 Si6	-1.7869	-0.632	0.0288 Si
7 O7	-1.383	-2.1502	-0.1041 O
8 O8	-2.9832	0.2815	-0.1772 O
9 O9	2.2861	-1.641	-0.1711 O
10 Mg10	0.52	-1.9599	0.0757 Mg
11 H11	-1.8032	2.0664	-0.5164 H

Table S22 Atomic Positions (MOL2) of the Chiral Dimer + H – Structure J3

1 Si1	0.1397	1.6191	-0.1185 Si
2 O2	-0.2722	0.0725	-0.4965 O
3 O3	-0.9838	2.6125	0.1389 O
4 O4	1.7146	1.6992	-0.0357 O
5 Mg5	2.6596	0.0968	0.0901 Mg
6 Si6	-1.8705	-0.6496	-0.3849 Si
7 O7	-1.405	-2.1145	0.0487 O
8 O8	-2.677	0.2776	0.6885 O
9 O9	2.2619	-1.6408	0.2325 O
10 Mg10	0.4726	-1.9344	0.0622 Mg
11 H11	-2.4639	1.2249	0.6085 H

Table S23 Atomic Positions (MOL2) of the Chiral Dimer + H – Structure J4

1 Si1	0.6335	1.587	-0.1194 Si
2 O2	-0.3525	0.2516	-0.194 O
3 O3	0.0706	2.9678	-0.3807 O
4 O4	2.1015	1.0849	0.1837 O
5 Mg5	2.6876	-0.6603	0.3005 Mg
6 Si6	-2.1198	0.1464	-0.1591 Si
7 O7	-1.8878	-1.6056	-0.5478 O
8 O8	-2.7904	0.3142	1.2096 O
9 O9	1.7502	-2.1701	0.0801 O
10 Mg10	0.0072	-1.749	-0.183 Mg
11 H11	-2.6613	-2.1227	-0.3177 H

Table S24 Atomic Positions (MOL2) of the Chiral Dimer + H – Structure J5

1 Si1	-0.0246	1.6009	-0.0001 Si
2 O2	1.4456	0.9343	-0.0002 O
3 O3	-0.3288	3.0811	-0.0002 O
4 O4	-1.125	0.4232	0.0001 O
5 Mg5	-3.1054	0.1632	0.0003 Mg
6 Si6	2.2169	-0.5264	-0.0002 Si
7 O7	1.1276	-1.6726	0.0000 O
8 O8	3.7276	-0.5888	-0.0003 O
9 O9	-2.5719	-1.76	0.0004 O
10 Mg10	-0.7059	-1.4854	0.0002 Mg
11 H11	-3.1564	-2.5132	0.0005 H

Table S25 Atomic Positions (MOL2) of the Chiral Dimer + H – Structure J6

1 Si1	-0.5623	-1.6269	-0.2589 Si
2 O2	0.4307	-0.2647	-0.1562 O
3 O3	-0.5376	-2.3994	1.2156 O
4 O4	-2.0631	-1.1196	-0.4463 O
5 Mg5	-2.5987	0.614	-0.1395 Mg
6 Si6	2.0556	0.1236	-0.0819 Si
7 O7	1.9447	1.6807	0.161 O
8 O8	3.1451	-0.9127	-0.2452 O
9 O9	-1.7309	2.1522	0.1556 O
10 Mg10	0.0648	1.9225	0.1907 Mg
11 H11	-0.0095	-2.4852	-1.3182 H

Table S26 Atomic Positions (MOL2) of the Chiral Dimer + H – Structure J7

1 Si1	0.6435	1.6364	-0.0819 Si
2 O2	-0.3217	0.2958	-0.1498 O
3 O3	0.0526	3.0225	-0.2292 O
4 O4	2.1269	1.1275	0.1207 O
5 Mg5	2.6024	-0.657	0.2028 Mg
6 Si6	-1.996	-0.0454	-0.2595 Si
7 O7	-1.9095	-1.6355	-0.321 O
8 O8	-2.7789	0.4338	1.1226 O
9 O9	1.7165	-2.2056	0.0896 O
10 Mg10	-0.069	-1.9523	-0.1111 Mg
11 H11	-2.5506	0.73	-1.3824 H

Table S27 Atomic Positions (MOL2) of the Chiral Dimer + H^+ – Structure K

1 Si1	0.2455	1.6699	-0.0000 Si
2 O2	-0.4828	0.1593	-0.0000 O
3 O3	-0.4998	2.9739	-0.0001 O
4 O4	1.7913	1.3034	-0.0000 O
5 Mg5	2.9773	-0.0373	0.0001 Mg
6 Si6	-2.0877	-0.5139	-0.0000 Si
7 O7	-1.6002	-2.0211	0.0000 O
8 O8	-3.3296	0.3300	-0.0001 O
9 O9	2.1841	-1.7575	0.0001 O
10 Mg10	0.2416	-1.7541	0.0001 Mg
11 H11	2.6590	-2.5907	0.0002 H

Table S28 Atomic Positions (MOL2) of the Chiral Dimer + H_2 – Structure L1

1 Si1	-1.2168	1.7411	0.0000 Si
2 O2	0.0000	0.5731	0.0000 O
3 O3	-0.9752	3.2309	0.0000 O
4 O4	-2.4763	0.7858	0.0000 O
5 Mg5	-1.7476	-0.9490	0.0000 Mg
6 Si6	1.6624	0.4291	0.0000 Si
7 O7	1.9649	-1.1205	0.0000 O
8 O8	2.5795	1.6295	0.0000 O
9 O9	-0.9938	-2.5900	0.0000 O
10 Mg10	0.7972	-2.5535	0.0000 Mg
11 H11	1.9021	-4.4599	0.0000 H
12 H12	2.4713	-3.9642	0.0000 H

Table S29 Atomic Positions (MOL2) of the Chiral Dimer + H_2 – Structure L2

1 Si1	1.9613	-0.4629	0.0394 Si
2 O2	0.4093	0.1821	-0.0441 O
3 O3	3.2004	0.3546	0.3146 O
4 O4	1.6085	-1.9934	-0.1427 O
5 Mg5	-0.2735	-2.0256	-0.1724 Mg
6 Si6	-0.3677	1.6567	-0.1424 Si
7 O7	-1.8865	1.3622	0.1854 O
8 O8	0.3345	2.8984	-0.6415 O
9 O9	-2.0753	-1.9573	-0.0341 O
10 Mg10	-2.6959	-0.2929	0.1766 Mg
11 H11	0.5206	2.401	1.9492 H
12 H12	1.9613	-0.4629	0.0394 H

Table S31 Atomic Positions (MOL2) of the Chiral Dimer + H_2 – Structure L3

1 Si1	1.9197	-0.4332	-0.1501 Si
2 O2	0.3658	0.2213	-0.0729 O
3 O3	3.1463	0.3529	-0.5464 O
4 O4	1.5601	-1.9523	0.1025 O
5 Mg5	-0.3187	-1.9842	0.1412 Mg
6 Si6	-0.3974	1.699	0.0138 Si
7 O7	-1.9379	1.4047	-0.1854 O
8 O8	0.3539	2.9759	0.3108 O
9 O9	-2.1246	-1.9122	0.0711 O
10 Mg10	-2.7493	-0.25	-0.1396 Mg
11 H11	2.098	-0.0814	2.404 H
12 H12	2.497	0.4481	2.0485 H

Table S32 Atomic Positions (MOL2) of the Chiral Dimer + H_2 – Structure L4

1 Si1	-1.8062	-0.5926	0.0001 Si
2 O2	-0.32070	0.19800	0.00010 O
3 O3	-3.14640	0.10150	0.00030 O
4 O4	-1.30400	-2.09160	-0.00010 O
5 Mg5	0.56950	-1.99760	-0.00020 Mg
6 Si6	0.32910	1.73470	0.00020 Si
7 O7	1.89730	1.53840	-0.00010 O
8 O8	-0.52540	2.97990	0.00030 O
9 O9	2.36020	-1.75990	-0.00030 O
10 Mg10	2.84600	-0.03910	-0.00030 Mg
11 H11	-6.37050	0.38200	-0.00070 H
12 H12	-5.62430	0.33910	0.00060 H

S.4 References

(1) Valencia, E. M.; Worth, C. J.; Fortenberry, R. C. Enstatite (MgSiO3) and forsterite (Mg_2SiO_4) monomers and dimers: highly detectable infrared and radioastronomical molecular building blocks. *Monthly Notices of the Royal Astronomical Society* **2020**, *492*(1), 276-282.

(2) Janesko, B. G. Topological analysis of the electron delocalization range. *Journal of Computational Chemistry* **2016**, *37*(21), 1993-2005.

(3) Hu, Y.; Chaka, A.; Dixon, D. A. Thermodynamics of the Metal Carbonates and Bicarbonates of Mn, Co, Ni, Cu, and Zn Relevant to Mineral Energetics. *The Journal of Physical Chemistry A* **2022**, *126*(43), 7874-7887.

(4) Hu, Y., Vasiliu, M.; Thanthiriwatte, K. S.; Jackson, V. E.; Chaka, A. M.; Dixon, D. A. Thermodynamics of metal carbonates and bicarbonates and their hydrates for Mg, Ca, Fe, and Cd relevant to mineral energetics. *The Journal of Physical Chemistry A* **2020**, *124*(9), 1829-1840.

(5) Reber, A. C., Paranthaman, S., Clayborne, P. A., Khanna, S. N., and Castleman Jr, A. W. From SiO molecules to silicates in circumstellar space: Atomic structures, growth patterns, and optical signatures of Si_nO_m clusters. *ACS Nano* **2008**, *2*(8), 1729-1737.

(6) Watson, W. D. The rate of formation of interstellar molecules by ion-molecule reactions. *Astrophysical Journal* **1973***, 183, L17.*

(7) Herbst, E.; Klemperer, W. The formation and depletion of molecules in dense interstellar clouds. *The Astrophysical Journal* **1973**, *185*, 505-534.

(8) Herbst, E. The astrochemistry of H_3^+. *Philosophical Transactions of the Royal Society of London. Series A: Mathematical, Physical and Engineering Sciences* **2000**, *358*(1774), 2523-2534.

(9) Agúndez, M.; Goicoechea, J. R.; Cernicharo, J.; Faure, A.; Roueff, E. The chemistry of vibrationally excited H_2 in the interstellar medium. *The Astrophysical Journal* **2010**, *713*(1), 662.
